# Self-healing and superstretchable conductors from hierarchical nanowire assemblies

**DOI:** 10.1038/s41467-018-05238-w

**Published:** 2018-07-17

**Authors:** Pin Song, Haili Qin, Huai-Ling Gao, Huai-Ping Cong, Shu-Hong Yu

**Affiliations:** 1 Anhui Province Key Laboratory of Advanced Catalytic Materials and Reaction Engineering, School of Chemistry and Chemical Engineering, Hefei University of Technology, Hefei, 230009 P.R. China; 2 Division of Nanomaterials and Chemistry, Department of Chemistry, Hefei National Laboratory for Physical Sciences at the Microscale, Collaborative Innovation Center of Suzhou Nano Science and Technology, CAS Center for Excellence in Nanoscience, University of Science and Technology of China, Hefei, 230026 P.R. China

## Abstract

It is still a great challenge to improve deformability and fatigue-resistance of stretchable conductors when maintaining their high-level conductivity for practical use. Herein, a high-performance stretchable conductor with hierarchically ternary network and self-healing capability is demonstrated through in situ polymerizing N-isopropylacrylamide (NIPAM) on well-defined sulfur-containing molecule-modified Ag nanowire (AgNW) aerogel framework. Owing to hierarchical architecture from nanoscale to microscale and further to macroscale and strong interactions of polymer chains and AgNWs, the composite exhibits good conductivity of 93 S cm^−1^, excellent electromechanical stability up to superhigh tensile strain of 800% and strong fatigue-resistant ability through well accommodating the applied deformations and sharing external force in the network. Furthermore, the composite delivers a fast and strong healing capability induced by reversible Ag–S bonds, which enables the healed conductor to hold an impressive electromechanical property. These prominent demonstrations confirm this material as top performer for use as flexible, stretchable electronic devices.

## Abstract (web summary)

Stretchable conductors are important for further developments in the electronics industry, but improving the deformability when maintaining the high-level conductivity is still challenging. Here the authors demonstrate a ternary self-healing silver nanowire/polymer network as high-performance stretchable conductor.

## Introduction

Conductive and stretchable materials find diverse applications in the fields of stretchable displays^1^, high frequency antennas^2^, artificial muscles^3^, as well as skin sensors^4–6^. One-dimensional (1D) conductive nanomaterials, especially carbon nanotubes (CNTs) have attracted great interests for building stretchable conductors because of their unique structural features enabling to accommodate bending and stretching deformations. However, these CNT-based elastic conductors suffer from the fact that high concentrations are required to demonstrate high electrical conductivity^7–11^. Unfortunately, due to heavy aggregation, a significant decay in their conductivity was occurred when increasing their elasticity^7–9,11^.

As another promising alternatives, metal nanowires, especially AgNWs having excellent electrical conductivity and mechanical resilience have made great contributions to elastic conductors^12–17^. However, due to lack of crack energy dissipation, elastic conductors with randomly dispersed AgNWs can only endure small deformations and their electrical resistances change significantly under tensile force. To overcome this challenge, many efforts have been focused on assembling 1D conductive nanowires into three-dimensional (3D) architectures to maintain stable network structure under shape deformations^18–20^. It was reported that a binary-network structure was designed to enhance the electromechanical performance of polyurethane sponge-AgNW-poly(dimethylsiloxane) (PU-AgNW-PDMS) stretchable conductors through assembling AgNW network into 3D sponge skeleton^18^. In the following, a typical stretchable conductor was demonstrated through infiltrating 3D AgNW conductive aerogel framework with elastic PDMS substrate^21^. Based on unique network structure, the obtained AgNW/PDMS composite showed improved performance with resistance variation of 150% under tensile strain of 100%. Even so, because of structural defects in PDMS networks and weak interfaces between elastic substrate and metal nanowires, a high level of conductivity was not allowed under a large extension. A kind of highly stretchable conductive fibers consisting of AgNWs, Ag nanoparticles and elastic styrene–butadiene–styrene matrix showed a maximum break elongation of 900% through a wet-spinning method. However, the composite fiber lost its electrical connection at a strain larger than 220%^22^. In spite of these significant achievements in stretchable conductors, it is still facing great challenges in rationally designing and optimizing the architecture of the conductors and therefore, improving their deformability, electromechanical stability, and fatigue-resistance for real applications.

Polymeric hydrogels with cross-linked polymer chains and high content of water have been well-accepted as a kind of elastic materials system. One hot topic involved in hydrogels is to improve their weak mechanical properties arising from the irregular cross-linking points and broad distributions of polymer chains^23^. Double network hydrogels^24^ and nanocomposite hydrogels^23^ are two typical models with enhanced mechanical strength. For the former, the crack energy is effectively dissipated through deforming network conformation or sliding cross-link points along polymer chains^25,26^. As for nanocomposite hydrogels, the nanofillers as large cross-linkers attached with polymer chains can relax the applied stress and delay the crack propagation over the whole network^27–29^. Herein, these nanocross-linkers were individually dispersed in the hydrogels. In case of 3D conductive assembly architecture with double network as whole cross-linker, the obtained hydrogel would be attractive as stretchable conductors due to novel energy dissipation mechanism guaranteeing high electromechanical property. Moreover, self-healing is another important feature related with the lifetime and sustainability in the use of stretchable conductors when damaged. However, rare work has focused on this subject possibly due to great difficulty in simultaneous translation of high conductivity, large stretchability and self-healing performance into an ensemble.

Based on above concept, we demonstrate the construction of high-performance stretchable conductors based on AgNW aerogel-based ternary network (AATN) hydrogel by a combination of backfilling poly(N-isopropylacrylamide) (PNIPAM) into highly ordered AgNW aerogel and in situ polymerization strategy. As a first demonstration of serving the aerogel framework as an integrated, large cross-linker, AATN hydrogel presents a cellular architecture with three levels of hierarchy from nanoscopic to microscopic and further to macroscopic scales, which effectively prevent crack propagation over the entire hydrogel through deforming the cellular structure and sharing the external stress in the whole network. Furthermore, the deformation/reformation of dynamic Ag–S bonds in the framework helps to stabilize the network structure when loaded on a tensile stress. As the best performer among stretchable conductors, the AATN composite shows high conductivity as AgNW aerogel and excellent electromechanical property with a resistance variation of 20% at tensile strain of 100% and nearly negligibly irreversible resistance at strains from 100 to 800% with 500 cycles. In addition, the composite hydrogel delivers a fast and strong self-healing capability with the healing efficiency of 93% induced by the reversible Ag–S bonds under near-infrared (NIR) laser irradiation. More impressively, the healed hydrogel conductors still maintain excellent conductive and mechanical properties, which makes a good demonstration of high-performance stretchable conductors with unique self-healing capability.

## Results

### Preparation of superstretchable AATN hydrogel

**Fig. 1:**
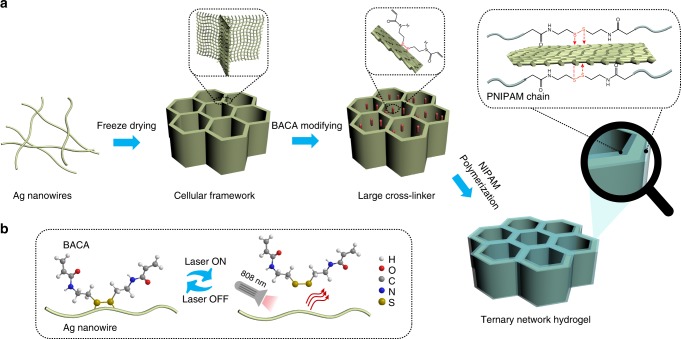
Struture design and self-healing mechanism. **a** Schematic illustrations of the preparation of AATN hydrogel. Typically, AgNW aerogel with cellular structure was prefabricated by the freeze-drying method. Being modified with BACA molecules, AgNW aerogel was used as large cross-linker for the synthesis of AATN hydrogel. **b** Schematics of the dynamic Ag-RS interaction between BACA and AgNWs under the NIR laser on/off

**Fig. 2:**
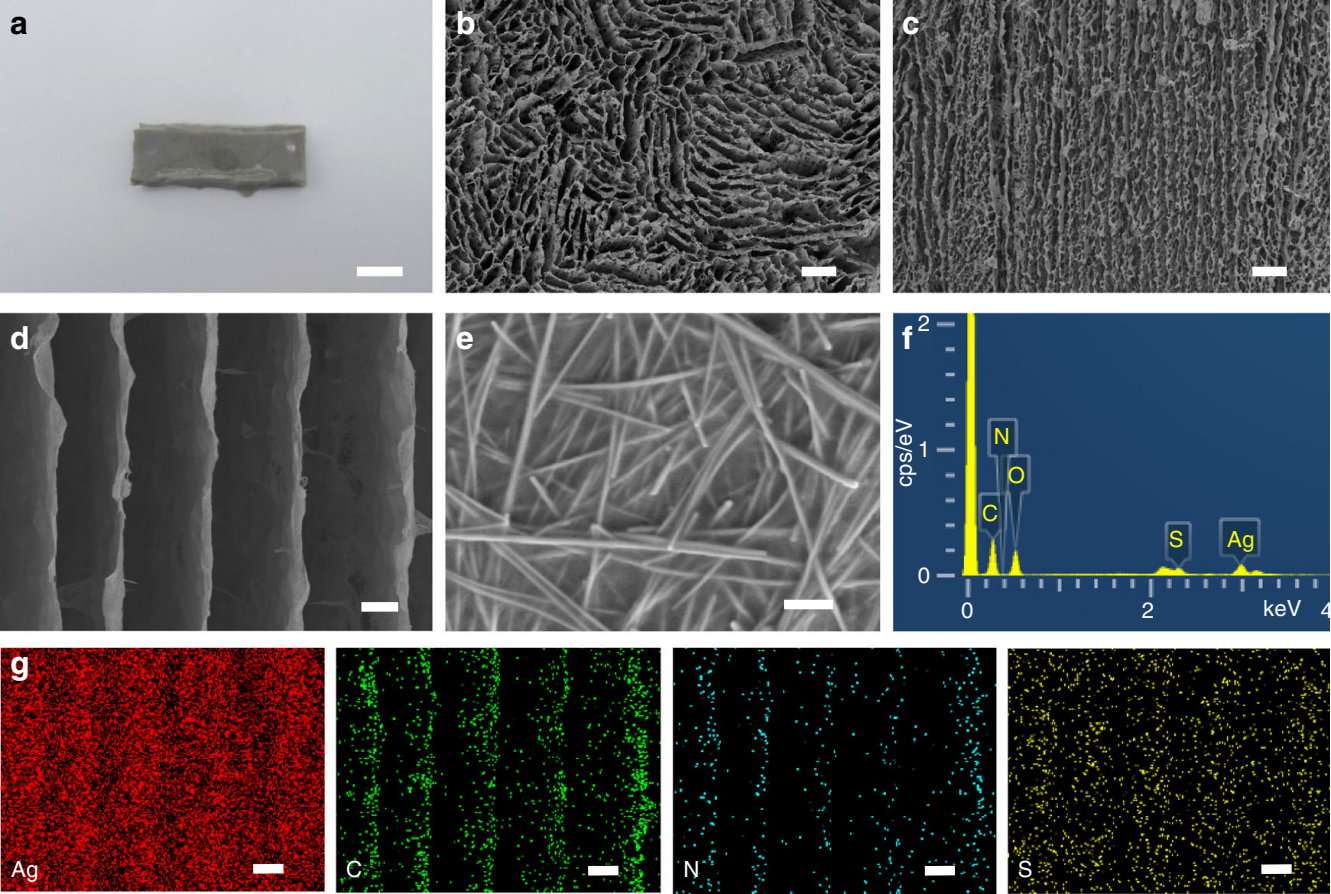
Structural and compositional characterizations. **a** Photograph of a piece of AATN hydrogel. Scale bar, 5 mm. **b** Top-view SEM image of AATN hydrogel. Scale bar, 100 μm. **c**, **d** Different magnifications of side-view SEM images of AATN hydrogel. Scale bars, 100 μm in **c** and 20 μm in **d**. **e** Further magnified SEM image of the lamella in AATN hydrogel. Scale bar, 500 nm. **f** EDS spectrum of AATN hydrogel. **g** Elemental mappings of Ag, C, N, and S elements in AATN hydrogel in **d**. Scale bars, 20 μm

Figure 1a schematically illustrated the fabrication of a highly conductive, stretchable AATN hydrogel through backfilling PNIPAM hydrogel into N,N’-bis(acryloyl)cystamine (BACA)-modified AgNW aerogel as framework and cross-linker. In brief, firstly, the binary-network AgNW aerogel was constructed by using high-quality AgNWs with the length of 4–15 μm as the assembly units (Supplementary Fig. 1) through the unidirectional freezing method^21^. The AgNW aerogel delivered a continuous, cross-linked 3D network structure composed of the interconnected, well-ordered two-dimensional (2D) AgNW films in parallel arrangement (Supplementary Fig. 2). With the cellular AgNW aerogel modified with the sulfur-containing BACA molecules as large cross-linker, the integrated AATN hydrogel was prepared through a vacuum filtration assisted polymerization process (Fig. 2a). As shown in top-view scanning electron microscopy (SEM) image in Fig. 2b, AATN hydrogel demonstrated an undisrupted cellular structure with the compartmental size of ~15 μm. Arising from the filling of PNIPAM, the cellular wall was denser compared with the original 3D AgNW aerogel (Supplementary Fig. 2c). Impressively, a highly ordered film array parallel to the growth of ice crystals was presented in the side-view SEM image in Fig. 2c, d. In further magnification, it was clearly investigated that the parallel lamella was composed of interconnected AgNWs coated with a thin layer of PNIPAM uniformly (Fig. 2e). Energy dispersive spectroscopy (EDS) analysis indicated Ag, C, N, S, and O elements in AATN hydrogel (Fig. 2f). Herein, the profile of S element was originated from BACA. Moreover, homogeneous spacial distributions of Ag, C, N, and S elements were detected in elemental mappings of AATN hydrogel, fully revealing its uniform wrapping of polymer on AgNWs (Fig. 2g). In addition, Fourier transform infrared spectroscopy (FT-IR) spectrum of AATN hydrogel showed the characteristic peaks of N–H bending, C = O stretching and CH_3_ asymmetric stretching of PNIPAM, which strongly verified incorporation of PNIPAM in AgNW aerogel (Supplementary Fig. 3)^30^. Based on above analysis, a hierarchically ternary network was formed in hydrogel, that is, at nanoscale, AgNWs were assembled into 2D Ag nanofilm arrays along the ice growth direction (first order), these Ag films as compartmental units were then interconnected with each other, forming a well-oriented cellular microstructure (second order), and under the strong Ag-thiolate (Ag-RS) coordinations, PNIPAM cross-linked with Ag conductive network enabled the success in building a robust macroscopic assembly (third order).

Similarly, a series of AATN hydrogels with well-defined top-view and side-view microstructures and varied compartmental sizes were constructed through backfilling PNIPAM into AgNW aerogels assembled with different contents of AgNWs or at varied freezing temperatures (Supplementary Figs. 4 and 5). It was reported that the microstructure of AgNW aerogels was tightly dependent on the configuration of ice crystals grown in the AgNW suspension, which was greatly influenced by the content of AgNW and freezing temperature^31^. When decreasing the freezing temperature, with the same amount of AgNWs, the 2D AgNW network films in the aerogel became much thinner and the number of compartments increased arising from more and smaller initial ice crystals (Supplementary Fig. 6). As such, well-defined AATN hydrogels with ordered structure were prepared through controlling the appropriate AgNW content or freezing temperature. With increasing AgNW content from 30 to 50 mg cm^−3^, the microstructures in network were more compact with the cellular size among the compartmental networks reduced from ~45 to ~22 μm (Supplementary Fig. 4). A similar trend in structural change of AATN hydrogels was observed when decreasing the freezing temperature arising from the formation of the smaller ice crystals at the lower temperature (Supplementary Fig. 5).

The electrical property of AgNW aerogels and AATN hydrogels was extensively studied with increasing the density of AgNWs or reducing the freezing temperature. It was measured that the obtained AgNW aerogel had high electrical conductivity of 97 S cm^−1^ with the density of 60 mg cm^−3^ (Supplementary Fig. 7). With further thermal annealing treatment in Ar/H_2_ (95%/5%) atmosphere at 230 °C for 2 h, its electrical conductivity was improved to 202 S cm^−1^ due to the removing of the polyvinyl pyrrolidone (PVP) coating and the nanowelding between the AgNWs^12, 32^, as confirmed by FT-IR (Supplementary Fig. 8) and transmission electron microscopy (TEM) analysis (Supplementary Fig. 9). Interestingly, according to percolation theory^33^, the power-law scaling well described the relationships between relative conductivity (*σ*/*σ*_s_, bulk Ag conductivity *σ*_s_ = 6.3 × 10^5^ S cm^−1^) and relative density (*ρ*/*ρ*_s_, bulk density *ρ*_s_ = 10.49 g cm^−3^) for both as made and sintered AgNW aerogels (Supplementary Fig. 10). The scaling exponent of sintered aerogel was even high to 2.79, consistent with that of the reported AgNW aerogel^32^ and CuNW aerosponge^34^, indicating good conductivity of AgNW components and good contact between them. Herein, balanced between the hydrophilicity and conductivity, the nonsintered AgNW aerogel was selected as the framework for the infiltration of PNIPAM. With decreasing the freezing temperature, the conductivity was improved obviously due to more electronic transport channels from the thinner and increased compartments in the 3D cellular structure with the same content of AgNWs (Supplementary Fig. 6).

Owing to the preserved highly ordered cellular structure, although AATN hydrogels were coated with a layer of PNIPAM, they still maintained high-level electrical conductivity as the 3D AgNW scaffolds with the conductivity in the range of tens of S cm^−1^. For example, the composite hydrogel with 60 mg cm^−3^ of AgNW aerogel delivered a conductivity of 93 S cm^−1^. In strong contrast, the PNIPAM hydrogel and the hydrogel incorporated with the randomly dispersed AgNWs (AgNW/PNIPAM) were nearly insulated with the resistance in kΩ order of magnitude for 20 mm × 6 mm × 2 mm sample (Supplementary Fig. 11), fully indicating the superiority of the assembled 3D AgNW architecture as the framework in significantly enhancing electrical conductivity of the obtained hydrogel. Based on the above quantitative analysis, the AATN hydrogel demonstrated its highly competitive potentials in the fields of the conductor compared with the reported pioneer work (Supplementary Table 1).

### Mechanical and electromechanical performances

**Fig. 3:**
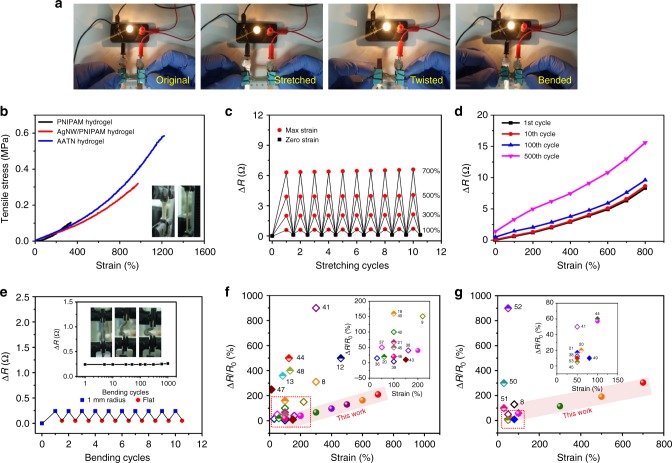
Mechanical and electromechanical performances. **a** Photographs demonstrating good electrical conductivity of AATN hydrogel when stretched, twisted and bended. **b** Tensile stress–strain curves of PNIPAM hydrogel (black line), AgNW/PNIPAM hydrogel (red line), and AATN hydrogel (blue line). The inset showing AATN hydrogel when stretched. **c** Variation of electrical resistance (Δ*R*) of AATN hydrogel during 10 stretching-releasing cycles with the fixed strains of 100, 300, 500, and 700%, respectively. **d** Δ*R* values of AATN hydrogel during 1st, 10th, 100th, and 500th cycle with a continuous stretching up to a strain of 800%. **e** Δ*R* values of AATN hydrogel during the bending-releasing cycles with a small bending radius of 1 mm. The inset showing the hydrogel during bending process and Δ*R* values during 1000 cycles. **f** Ashby chart plotting normalized resistance change (Δ*R*/*R*_0_) vs. tensile strain of AATN hydrogel and the reported stretchable conductors. The inset showing enlarged view of the rectangular area. Numbers stand for relevant references. (Hollow diamond: CNT-based stretchable conductor; solid diamond: graphene-based conductor; semi-hollow diamond: metal nanowire-based conductor; and solid sphere: AATN hydrogel). **g** Ashby chart plotting Δ*R*/*R*_0_ vs. tensile strain of AATN hydrogel and the reported stretchable conductors with 100 stretching cycles. The insert showing enlarged view of the rectangular area. Numbers stand for relevant references. (Hollow diamond: CNT-based conductor; semi-hollow diamond: metal nanowire-based conductor; solid diamond: other conductor; and solid sphere: AATN hydrogel)

For evaluating the practicability of AATN hydrogel as the stretchable conductor, its mechanical and electromechanical properties were studied in detail. As shown in Fig. 3a, the lighted lamp kept constant brightness with connecting a piece of AATN hydrogel even when the hydrogel was stretched, twisted or bended, indicating its stable network under varied mechanical deformations. The mechanical property of AATN hydrogel was quantified by the tensile stress–strain curve and the corresponding measurements were also carried out on the control samples of the conventional PNIPAM hydrogel and AgNW/PNIPAM hydrogel (Fig. 3b). For PNIPAM hydrogel, the tensile stress was only 0.10 MPa at the ultimate elongation of 330%. With the incorporation of AgNWs into PNIPAM, the mechanical performance of the obtained nanocomposite hydrogel was reinforced with the tensile strength of 0.32 MPa at the strain of 1030%. When the preassembled AgNW aerogel was used as a whole cross-linker in polymerization process, the AATN hydrogel delivered an impressive stretchability with significantly enhanced tensile stress of 0.60 MPa at the strain of 1230%, suggesting great contributions of highly ordered assembled architecture to the improved mechanical performance of the resulted hydrogel.

The compressive stress–strain tests were also conducted on the AgNW aerogels and AATN composites with different densities of AgNWs (Supplementary Fig. 12a, c). The compressive curves of these samples demonstrated typical behaviors of porous structures with well-shaped hysteresis loops within stains of 50%, namely elastic deformation followed by densification^19^. According to the slopes of these stress–strain curves within the initial linear elastic regime, relative Young’s modulus (*E*/*E*_s_, bulk Ag Young’s modulus *E*_s_ ≈ 70 GPa) as a function of relative density (*ρ*/*ρ*_s_) was plotted (Supplementary Fig. 12b, d). As for AgNW aerogel, *E*/*E*_s_ increased rapidly from 2.04 × 10^−7^ to 1.657 × 10^−6^ with the increasing of *ρ*/*ρ*_s_ from 2.86 × 10^−3^ to 5.72 × 10^−3^, revealing a quantitative scaling behavior with the scaling exponent of 2.86, suggesting a bending dominant deformation mechanism^32, 34^. However, combined with the coating of stretchable polymer chains, AATN materials delivered the scaling exponent of only 0.67, indicating a stretching dominant deformation^35^, which was in agreement with its high stretchability as shown in the tensile tests (Fig. 3b).

Given that AATN hydrogel possessed the combined merits of high electrical conductivity and excellent stretchability, we systematically investigated the variation of the electrical resistance with stretching deformations during the cycling process for further estimating its potentials as stretchable conductors. As shown in Fig. 3c, when the hydrogel was stretched to a fixed strain, its resistance was increased and could recover the initial value completely since releasing the tensile stress. Note that the hydrogel delivered a slight resistance variation (Δ*R*) of 0.6, 1.9, and 3.8 Ω with normalized resistance change (Δ*R*/*R*_0_) of 20, 67, and 130% at the tensile strains of 100, 300, and 500%, respectively. Even the hydrogel was stretched to a superhigh strain of 700%, a resistance change of only 6.2 Ω was indicated with the resistance recovered after 10 cycles (Fig. 3c). Such performance was much better than that of the previously reported stretchable conductors composed of various conductive constituents including CNTs, graphene, and metal nanowires in terms of maximum tensile strain and electromechanical stability (Fig. 3f, Supplementary Table 2)^8,9,12,13,18, 20, 21, 36–48^. Then, the durability of its electromechanical performance under the continuous stretching was further carried out by using a mechanical test system. With elongating the hydrogel, its resistance was increased continually until reaching the conductive limit with the strain more than 800% as observed in Fig. 3d. Moreover, during the first 100 stretching cycles with the strains from 100 to 800%, the profiles of resistance variation were highly constant with a negligibly irreversible resistance, which confirmed AATN hydrogel as one of the top-performers in the applications of stretchable conductors (Fig. 3g, Supplementary Table 3)^8,20,21,38,41,44, 45,49–53^. Even after 500 cycles, the irreversible resistance was only 1.4 Ω and a reasonably increased fluctuation of the resistance change was shown, for example, Δ*R* increased from 2.0 to 6.1 Ω at the strain of 300%, illustrating highly stable 3D network structure in the hydrogel. Compared with the previously reported work, AATN hydrogel was the best performer for use as stretchable conductors based on the combined merits of high electromechanical stability, large deformability and strong fatigue-resistant capability (Fig. 3f, g, Supplementary Tables 2 and 3).

Furthermore, the resistance variation of the AATN hydrogel under bending deformations was also investigated in Fig. 3e. It was noticed that there was only a slight increase of 0.24 Ω in resistance at the bending radius of 1.0 mm during the first cycle. When the hydrogel was straightened, its resistance was almost recovered to the original value. Even after 1000 bending-releasing cycles, the resistance variation was less than 0.26 Ω for such a small bending radius (Inset in Fig. 3e). All these results indicated its stable electromechanical performance due to the hierarchical network structure in the hydrogel.

### Microstructural analysis

**Fig. 4:**
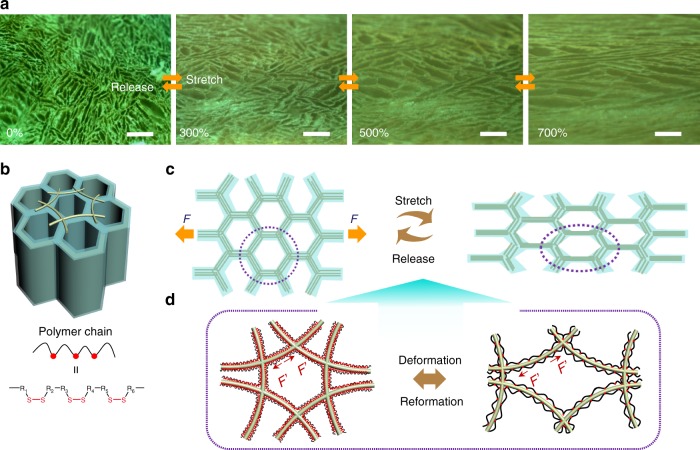
Microstructural analysis on high electromechanical stability under large stretch. **a** Optical images of the microstructure evolution of AATN hydrogel when stretched continuously with a strain of 0%, 300, 500, and 700%. With releasing the force, the structures can be recovered. Scale bars, 100 μm. **b** Schematics of the microstructure of AATN hydrogel. **c** Schematics of the elongated network of AATN hydrogel when loaded on a tensile stress and recovered structure since released. **d** Detailed schematics of the deformation and reformation of the dynamic coordination interaction between AgNWs and polymer chains in hydrogel during stretching and releasing process

In order to understand the function of the ternary network structure in bringing about the excellent performance of the AATN hydrogel, we further analyzed the microstructure evolution of the hydrogel under continuous tensile strain with the help of optical microscopy. As investigated in Fig. 4a, when loaded with the tensile force at the tensile strains from 0 to 700%, the compartmental units composed of AgNWs were still integrated with elongated parallel to the direction of the force and shrank in the perpendicular direction of the force, which was also shown from the cross-sectional SEM images of the fractured hydrogels during the stretching process with different strains (Supplementary Fig. 13). Herein, the AATN hydrogel composed of binary-networked AgNW aerogel interconnected with polymer chains can be regarded as a whole cross-linked network with AgNW aerogel as the large cross-linker. On the one hand, the shape deformation in the 3D framework was elastic and effectively relaxed the locally applied force. As the loading was removed, the network recovered to the initial state correspondingly as schematically demonstrated in Fig. 4b, c. No noticeable changes of the microstructure were observed in the optical images during the cycling tests with the strains from 0 to 700% and 20 stretching-releasing cycles with a fixed strain of 500%, respectively (Fig. 4a and Supplementary Fig. 14). On the other hand, the polymer chains cross-linked with AgNWs arising from the interactions of Ag–S coordination served as the crack bridge greatly increased the crack propagation resistance, which stabilized the deformation and protected the network from further damage. This dynamic coordination interaction between AgNWs and polymer chains brought about efficient energy transfer by bond deformation and reformation during the stretch, leading to a smaller strain on Ag network than polymer network (Fig. 4d)^25, 54^.

Additionally, owing to the unique, integrated cross-linker of AgNW aerogel architecture, the external stress (*F*) loaded on the hydrogel could be effectively spread over the whole network and delay the crack propagation. This avoided the local stress loading on the single nanowire or polymer chain and also reduced the force strength greatly through sharing the force in the whole network. Therefore, as shown in Fig. 4d, the resultant force (*F′*) applied on the building block was much smaller than *F*, maintaining the network of integrity effectively. With further applying the force on the hydrogel, the nanowires as crack bridges were pulled out of the hydrogel skeleton under the persistent stretching (Supplementary Fig. 15), another effective way to buffer and dissipate crack energy for preventing the crack propagation and stabilizing the architecture^55^. Moreover, the tightly interconnected AgNWs in each compartmental wall enabled a good accommodation of the stretching deformation without fading the electrical conductivity. In contrast, as for the pure AgNW aerogel without PNIPAM, its network was easily collapsed under a small tensile stress due to the lack of effective mechanism for the force relaxation and energy dissipation (Supplementary Fig. 16), indicating the superiority of conductive framework as unique cross-linker in achieving high electromechanical performance. In this way, such unique nano/microstructures in AATN hydrogel guaranteed a continuously and highly conductive network under large tensile strains.

### Self-healing performance

**Fig. 5:**
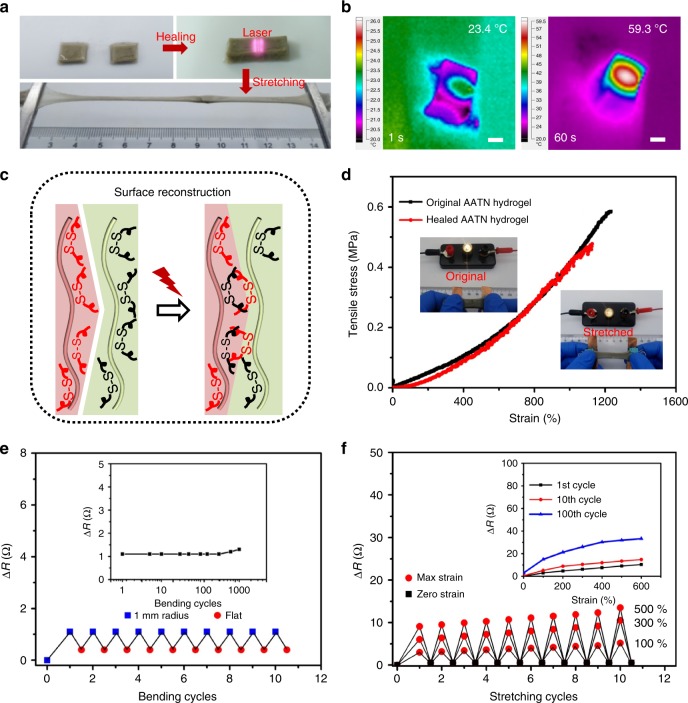
Self-healing performance. **a** Photographs showing NIR laser-induced healing process of the separated hydrogels. The healed sample presented high stretchability. **b** Temperature changes of AATN hydrogel with 60 s of NIR laser irradiation. Scale bars, 1 cm. **c** Schematic illustrations of healing mechanism of the AATN hydrogel through the dynamic Ag–S interaction induced surface reconstruction under the laser. **d** Tensile stress–strain curves of the original and healed AATN hydrogels. The inset showing the healed AATN hydrogel lightening up the lamp under stretched deformation. **e** Electrical resistance change (Δ*R*) of the healed AATN hydrogel during bending-releasing cycles with the bending radius of 1 mm. The inset showing Δ*R* values in 1000 bending cycles. **f** Δ*R* values of the healed AATN hydrogel during ten stretching-releasing cycles with the fixed strains of 100, 300, and 500%. The inset showing Δ*R* values of the healed hydrogel during 1st, 10th, and 100th cycles under continuous stretching

In consideration of the dynamic coordinate interactions of Ag-RS in the AATN hydrogel^56^, its self-healing performance was studied under the NIR laser. As shown in the photograph in Fig. 5a, when the two separated pieces of hydrogel were in close contact and exposed to the NIR laser, they were able to self-heal naturally in less than 1 min. The healed sample was still highly stretchable as the photograph illustrated. Herein, good photothermal effect of AgNWs arising from the surface plasmon resonance resulted in a fast temperature increase in AATN hydrogel upon the irradiation of NIR laser^57, 58^. As tested by the infrared imager in Fig. 5b, the temperature of the fractured joints of the hydrogel was increased from 23.4 to 59.3 °C in 1 min stimulated by NIR laser. In contrast, PNIPAM hydrogel without AgNWs was not heated, keeping the room temperature (Supplementary Fig. 17). As schematically illustrated in Fig. 1b, owing to the surface modification of AgNWs with sulfur-containing BACA molecules, such a fast and high temperature effectively triggered the dynamic Ag–RS on/off switching from the surface of the hydrogel^59^, therefore, leading to the surface reconstruction of Ag–RS bonds and the followed healing of AATN hydrogel (Fig. 5c)^60^. In the following, the mechanical property of the healed hydrogel was tested in quantity by using tensile stress–strain curve in Fig. 5d. The mechanical profile of the healed sample delivered a nearly overlapped path with that of the original hydrogel. Moreover, the recovered extensibility of the hydrogel reached 12 times of its initial length with the healing efficiency of nearly 93%, fully indicating its excellent self-healing capability. In addition, a set of AATN hydrogels with different AgNW contents all demonstrated good healing performance with the efficiency of ~90% (Supplementary Fig. 18 and Supplementary Table 4).

Impressively, the healed AATN hydrogel still displayed high electromechanical performance as revealed from the bright lamp when connected with the healed piece under the stretched, twisted and bended deformations (Inset in Fig. 5d and Supplementary Fig. 19). Its resistance variation under bending deformation was tested at the bending radius of 1 mm (Fig. 5e). In the first cycle, the hydrogel showed a resistance increase of 1.0 Ω and a slight irreversible resistance of 0.38 Ω when straightened. Since the second cycle, Δ*R* value was highly constant with the recovered value during bending-flatting process. With 1000 cycles, the resistance increase was only 1.3 Ω. Except for good performance at bending deformation, the healed hydrogel also showed impressive conductive behavior under tensile stress. At the tensile strain of 100%, the resistance variation was 3.0 Ω and could recover to the initial value when releasing (Fig. 5f). In the following ten cycles, the slightly irreversible resistance was observed. Similar results were measured for the healed sample at the strains of 300 and 500%. With extending the elongations, the resistance change was increased. In addition, the healed hydrogel piece could endure long-term cycling with the tensile strain ranging from 0 to 600%. In the 100th cycle, the resistance of the hydrogel was still increased smoothly until stretched to a large strain of 600% (Inset in Fig. 5f). These good electrical and mechanical performances fully proved high healing capability of the hydrogel and demonstrated its effectiveness as stretchable conductor in practical use.

## Discussion

In conclusion, we have successfully demonstrated the construction of AATN hydrogels as stretchable conductors with hierarchically ternary network structure through infiltrating PNIPAM hydrogel into preassembled AgNW aerogel. With the merits of well-organized architecture from nanoscopic to microscopic and further to macroscopic scales and dynamic Ag–RS coordination interaction in the framework, AATN hydrogel well accommodated the stretching deformations and showed high electromechanical performance through effectively relaxing the applied force and dissipating the crack energy. The structural advantage enabled AATN hydrogel as the best performer in the fields of stretchable conductors by delivering a slight resistance variation of only 20% at a strain of 100%, and nearly negligibly irreversible resistance at strains from 100 to 800% with 500 stretching cycles. Furthermore, the composite hydrogel exhibited a fast and strong self-healing capability with the healing efficiency of 93% induced by the reversible Ag–RS bonds, which contributed to an impressive electromechanical performance for healed hydrogel. The strategy presented in this work has demonstrated its effectiveness in design and construction of superstretchable conductor with excellent healing capability, which may find promising applications in flexible, stretchable electronic devices.

## Methods

### Preparation of the AgNWs

Firstly, 5.86 g of PVP and 190 mL of glycerol were added into a 500 mL round bottom flask. PVP was dissolved in the microwave. When the solution was cooled to the room temperature, 1.58 g of AgNO_3_ powder was added followed by addition of 10 mL of glycerol solution containing 59 mg of NaCl and 0.5 mL of H_2_O. With gently stirring, the solution was heated to 210 °C in 30 min. The color of the reaction solution was evolved from pale white to light brown, red, dark gray, and eventually grayish-green.

### Preparation of AATN hydrogels

Typically, a series of AgNW aerogels were firstly fabricated by the ice-template assembly of the above AgNW dispersion with different nanowire contents at different freezing temperatures. Then, 5 mL aqueous solution containing 1 g of NIPAM as monomer, 2 mg of BACA as the sulfur cross-linking agent and 20 mg of K_2_S_2_O_8_ as initiator was degassed with nitrogen. In the following, 20 µL of N′,N′-tetramethylenediamine as catalyst was added into above solution. Finally, the mixture was filled into the prepared 3D AgNW aerogel assisted by vacuum filtration and was kept at room temperature for 48 h for the complete polymerization. For reference, PNIPAM hydrogel was prepared through a similar polymerization process in the absence of AgNW aerogel. Additionally, AgNW/PNIPAM nanocomposite hydrogel was also fabricated by using the same content of AgNWs rather than AgNW aerogel in the polymerization process.

### Materials characterization

SEM images, EDS analysis and elemental mappings of the freeze-dried samples were carried out on a scanning electron microscope (Zeiss Merlin Compact, Germany) equipped with an Oxford Inca energy instrument at an accelerating voltage of 5 kV. TEM images were made using a Hitachi H7700 transmission electron microscopy at an acceleration voltage of 120 kV. FT-IR spectra were collected by a Thermo Nicolet 6700 spectrometer from 4000 to 400 cm^−1^ at room temperature. Tensile tests and bend tests were carried out on the Instron 5965A at a crosshead speed of 10 mm min^−1^. For compression test, the samples with a diameter of 15 mm and a height of 10 mm were measured using the testing machine equipped with a 10 N load cell under a quasi-static strain rate of 0.1 mm min^−1^. The electrical conductivity was measured by using a four-probe method. In order to ensure the accuracy of the conductivity test, both ends of AgNW aerogel were coated with silver glue before backfilling the polymer mixture. During the tests of mechanical and electrical properties, water was sprayed on the surface samples to avoid moisture loss. For self-healing process, two pieces of the separated hydrogels were in close contact and NIR laser with a wavelength of 808 nm and the light spot area of 1 cm × 0.5 cm was irradiated on the close contact point. The entire experiment was conducted at room temperature. Infrared images were obtained on a Fluke Ti400 infrared imager. The self-healing efficiency was calculated based on the tensile stress–strain curves of the original and healed samples.

### Data availability

The data that support the findings of this study are available on request from the corresponding authors (H.-P.C. or S.-H.Y.).

## Electronic supplementary material

Supplementary Information
