# Supplementary material for: Self-healing and superstretchable conductors from hierarchical nanowire assemblies
Source: Nat Commun. 2018 Jul 17;9:2786. doi: 10.1038/s41467-018-05238-w (PMC6050250; doi:10.1038/s41467-018-05238-w)
Supplement: Supplementary file 1 — Supplementary Information [file 41467_2018_5238_MOESM1_ESM.pdf]

Supplementary Information

Self-healing and superstretchable conductors from hierarchical nanowire assemblies

Song et al.

Supplementary Figures

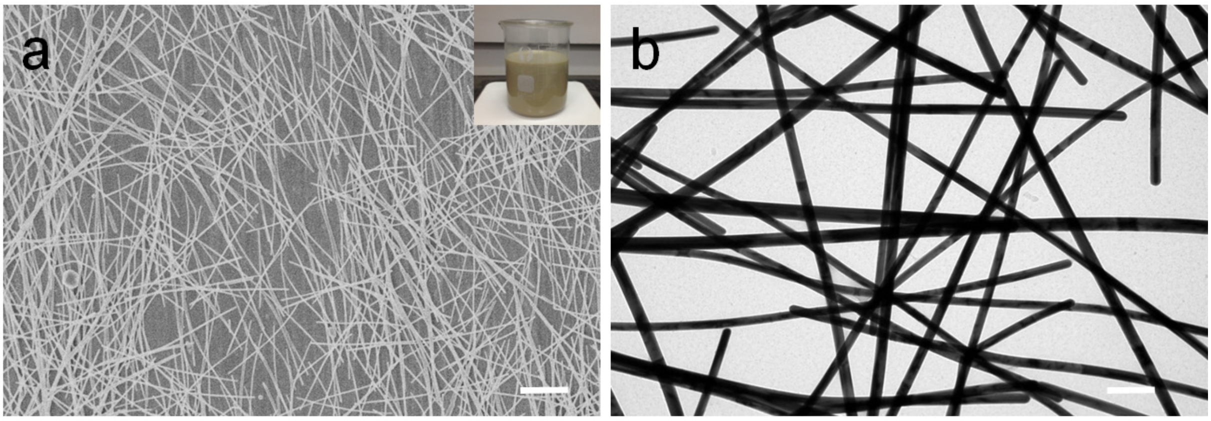

Supplementary Figure 1 | Characterization of AgNWs. a, SEM and **b,** TEM image of the prepared AgNWs. Inset in (**a**) showing the photograph of the dispersion of AgNWs. *Scale bars* in (**a**) 1 μm and in (**b**) 200 nm.

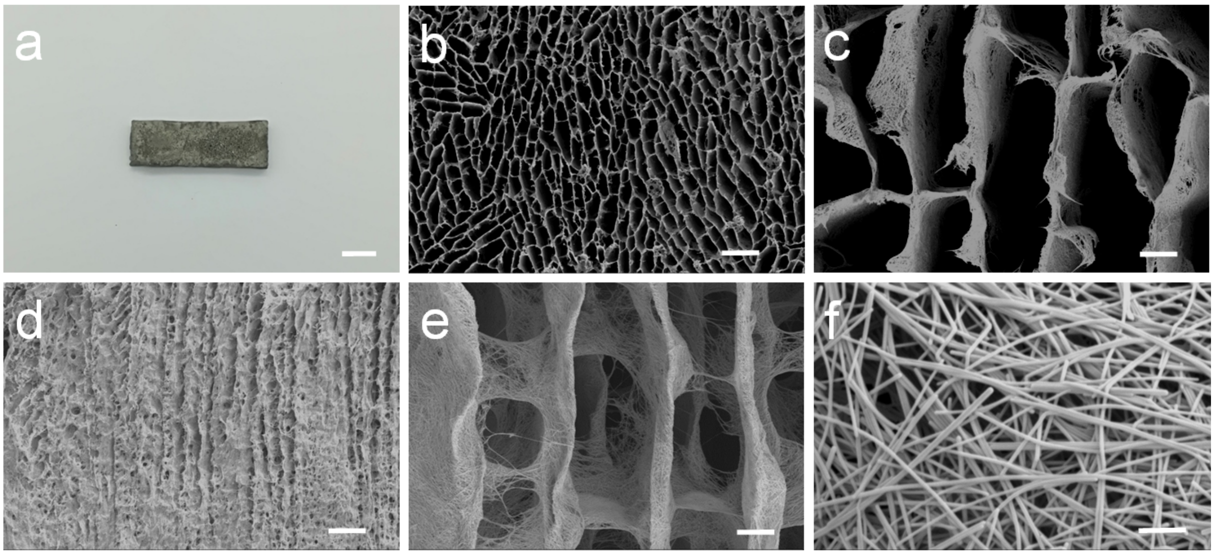

Supplementary Figure 2 | Characterization of AgNW aerogel. a, Photograph of the AgNW aerogel. *Scale bar*, 5 mm. **b,** Top-view SEM image of the AgNW aerogel. *Scale bar*, 100 μm. **c,** Magnified SEM image in (**b**). *Scale bar*, 10 μm. **d,** Side-view SEM image of the AgNW aerogel. *Scale bar*, 100 μm. **e, f,** SEM images with different magnifications in (**d**). *Scale bars* in (**e**) 10 μm and in (**f**) 500 nm.

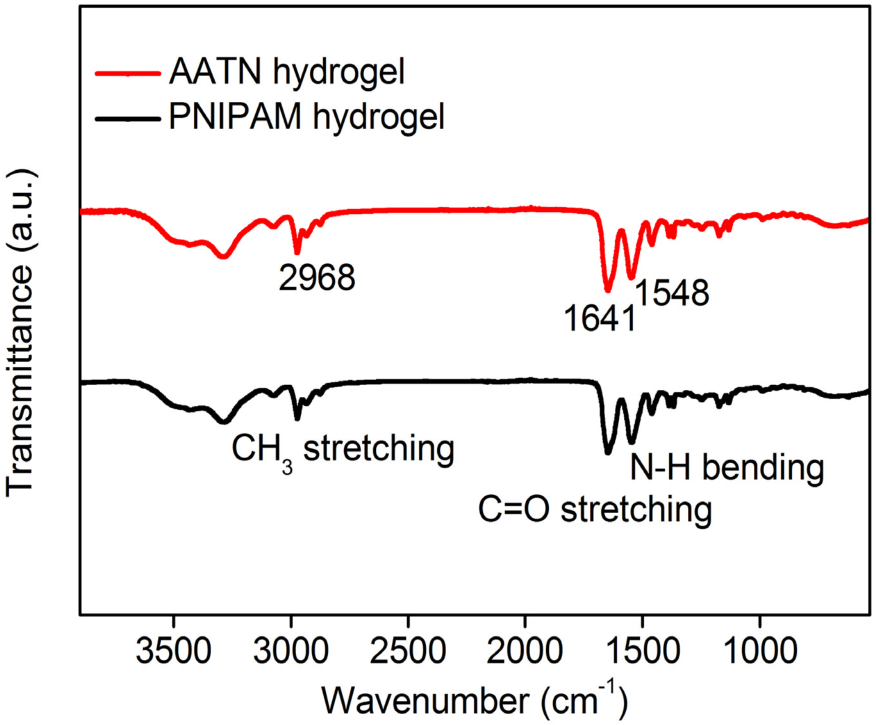

Supplementary Figure 3 | FT-IR spectra of AgNWs, PNIPAM hydrogel and AATN hydrogel.

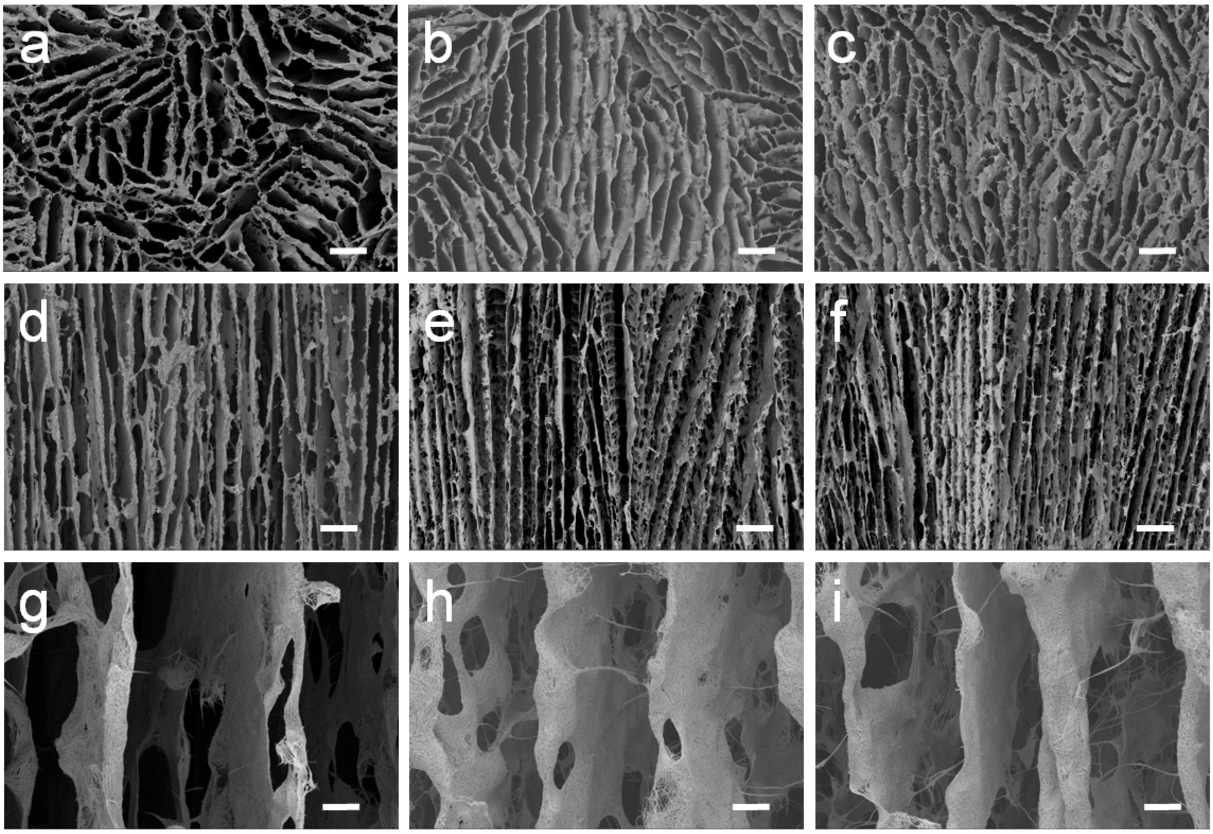

Supplementary Figure 4 | Microstructures of the AATN hydrogels. Top-view SEM images of the AATN hydrogels synthesized from AgNW aerogels freeze-dried at -30 ^{o}C with different contents of AgNWs: **a,** 30, **b,** 40 and **c,** 50 mg cm^{-3}. **d-f,** Corresponding side-view SEM images of the AATN hydrogels in (**a-c**). **g-i,** Magnified SEM images in (**d-f**). *Scale bars* in (**a-f**) 100 μm and in (**g-i**) 10 μm.

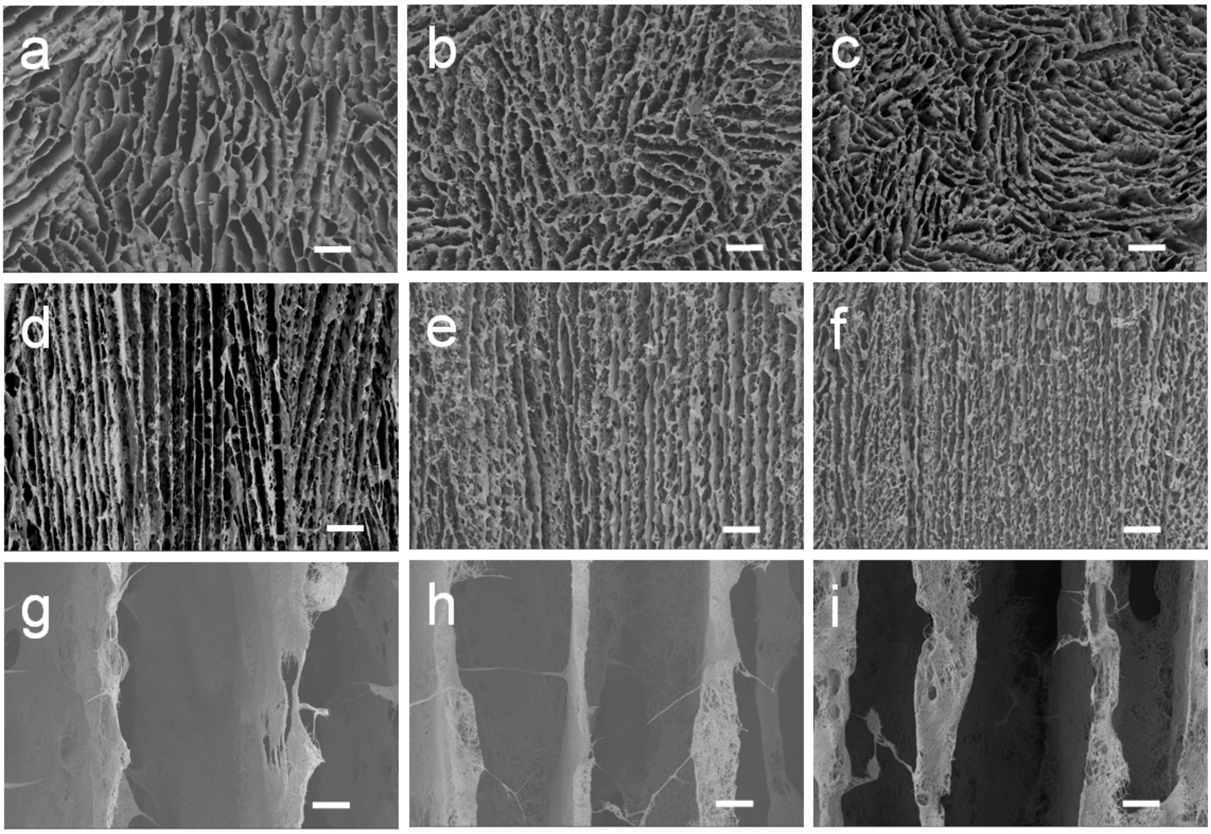

Supplementary Figure 5 | Microstructures of the AATN hydrogels. Top-view SEM images of the AATN hydrogels synthesized from 60 mg cm^{-3} of AgNW aerogels freeze-dried at different temperatures: **a,** -10 ^{o}C, **b,** -50 ^{o}C and **c,** -120 ^{o}C. **d-f,** Corresponding side-view SEM images of the AATN hydrogels in (**a-c**). **g-i,** Magnified SEM images in (**d-f**). *Scale bars* in (**a-f**) 100 μm and in (**g-i**) 10 μm.

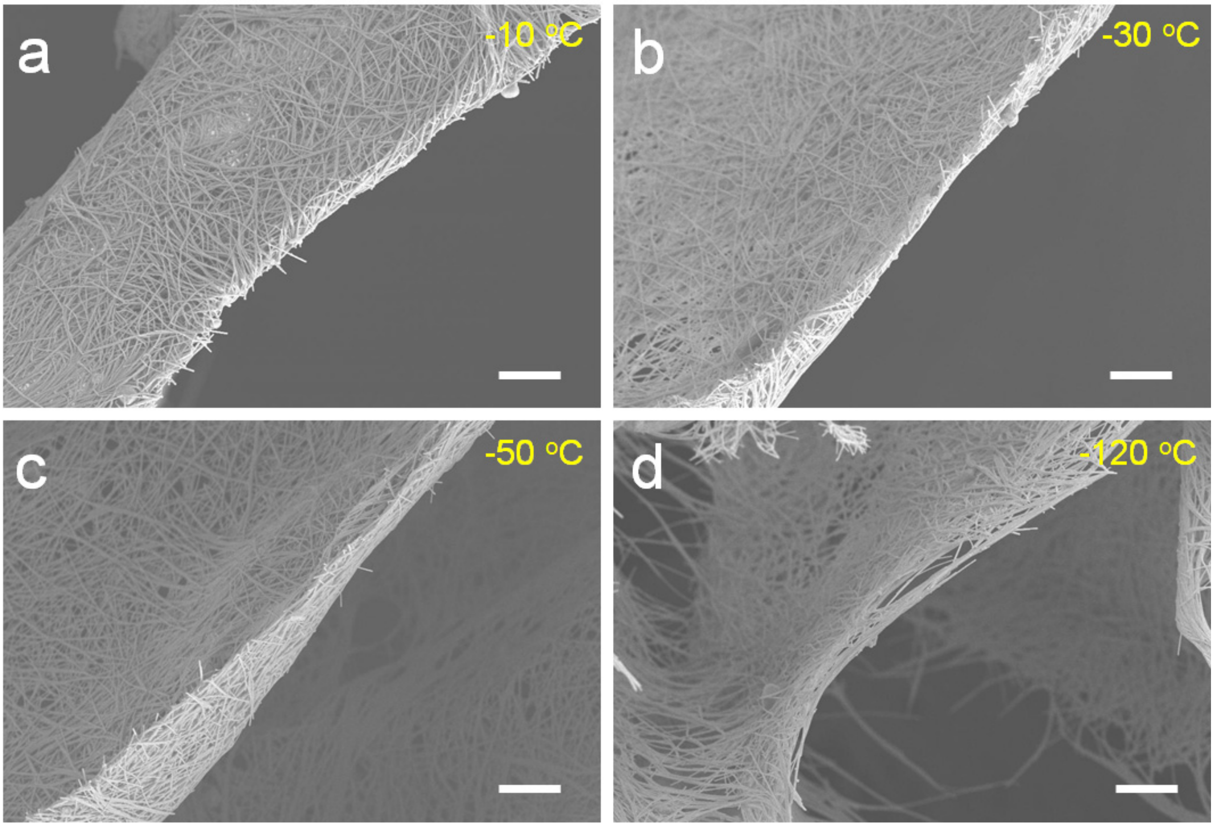

Supplementary Figure 6 | SEM images of AgNW aerogels prepared at different freezing temperatures. *Scale bars* in (**a-d**) 2 µm.

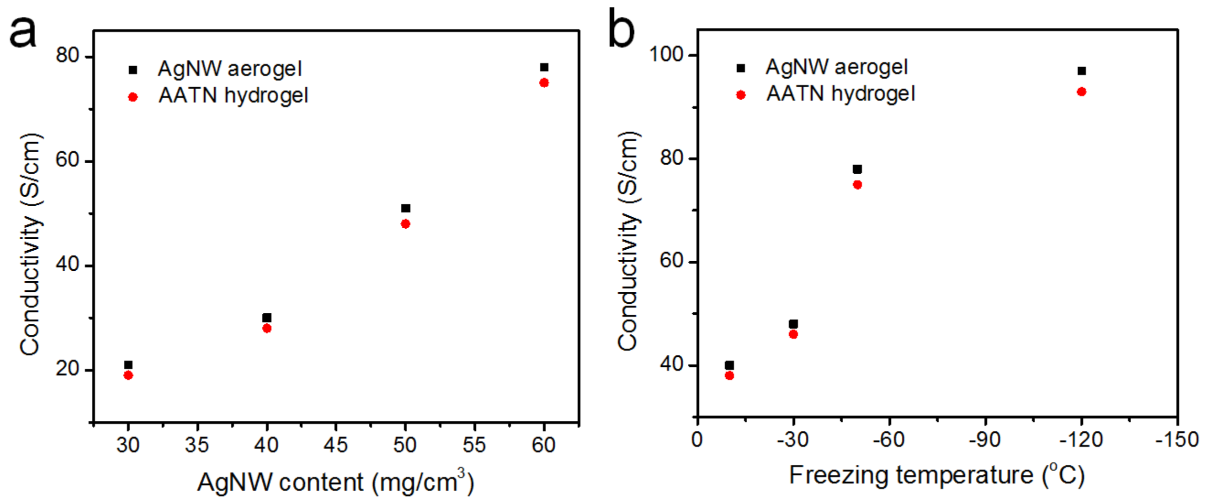

Supplementary Figure 7 | Electrical conductivity of AATN hydrogels and AgNW aerogels. a, Electrical conductivities of AATN hydrogels and AgNW aerogels prepared with different AgNW contents at -50 °C. **b,** Electrical conductivities of AATN hydrogels and AgNW aerogels prepared at different freezing temperatures with AgNW content of 60 mg cm^{-3}.

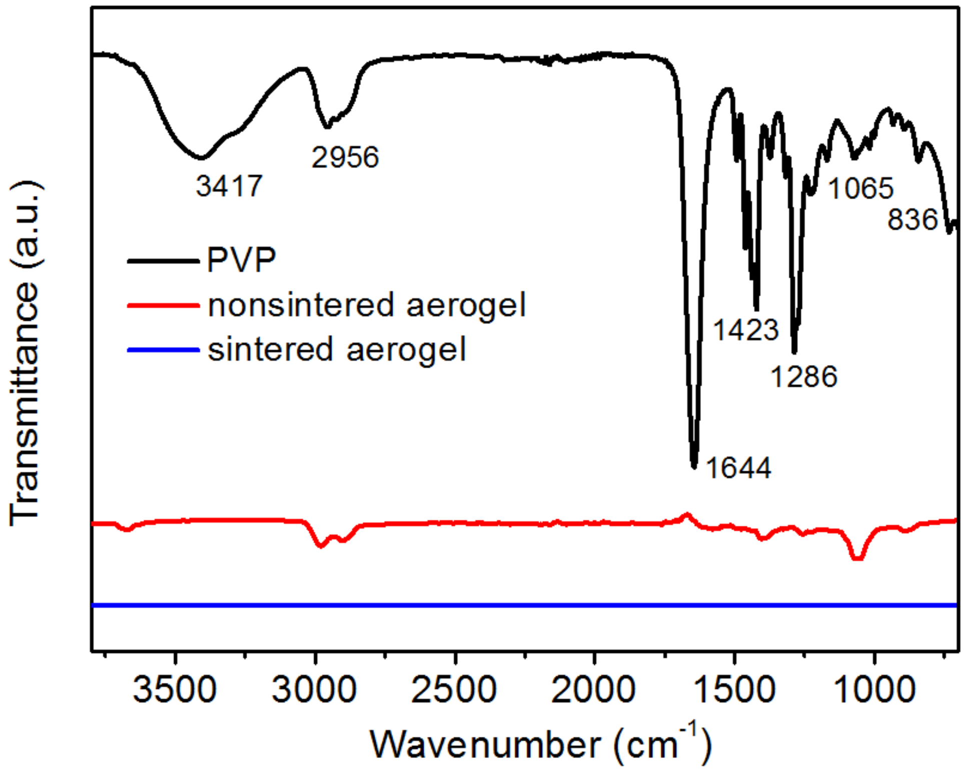

Supplementary Figure 8 | FT-IR spectra of PVP, nonsintered and sintered AgNW aerogels.

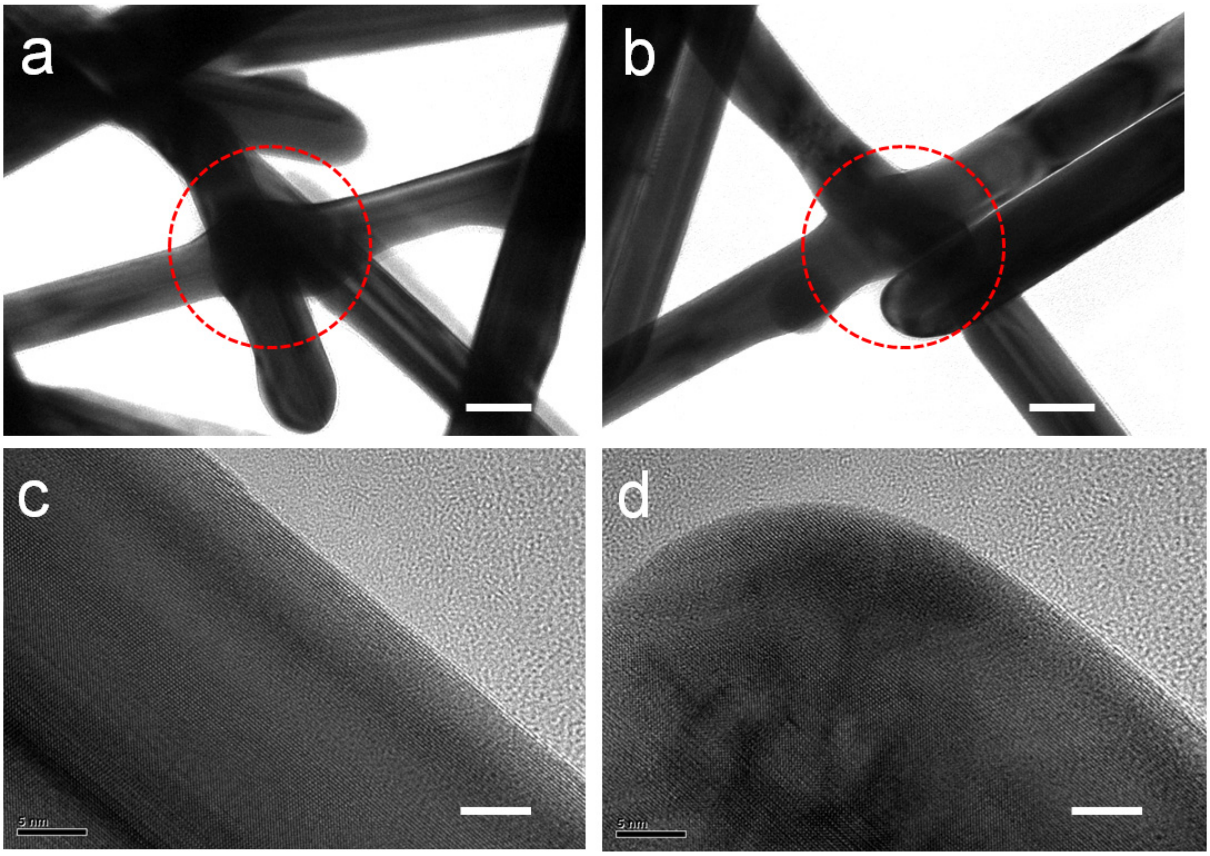

Supplementary Figure 9 | TEM images of sintered AgNW aerogel. *Scale bars* in (**a, b**) 50 nm and in (**c, d**) 5 nm.

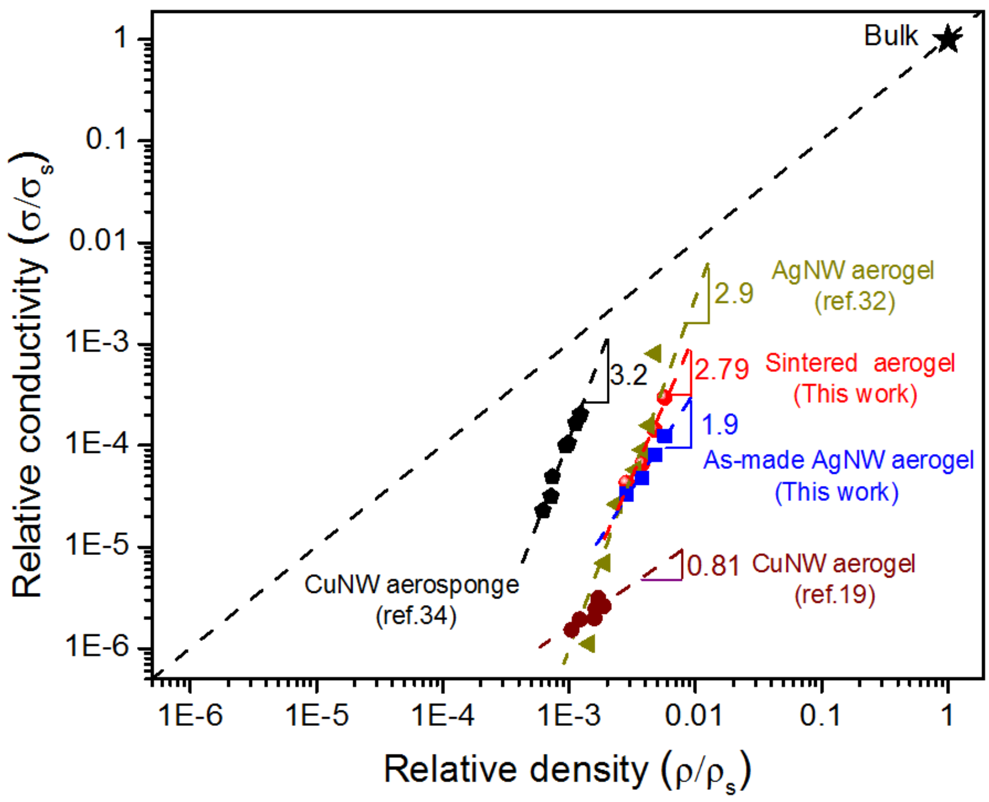

Supplementary Figure 10 | Electrical property of AgNW aerogels. The plot of relative conductivity (σ/σ_s) against relative density (ρ/ρ_s) of the as-made and sintered AgNW aerogels compared with other metal aerogels.

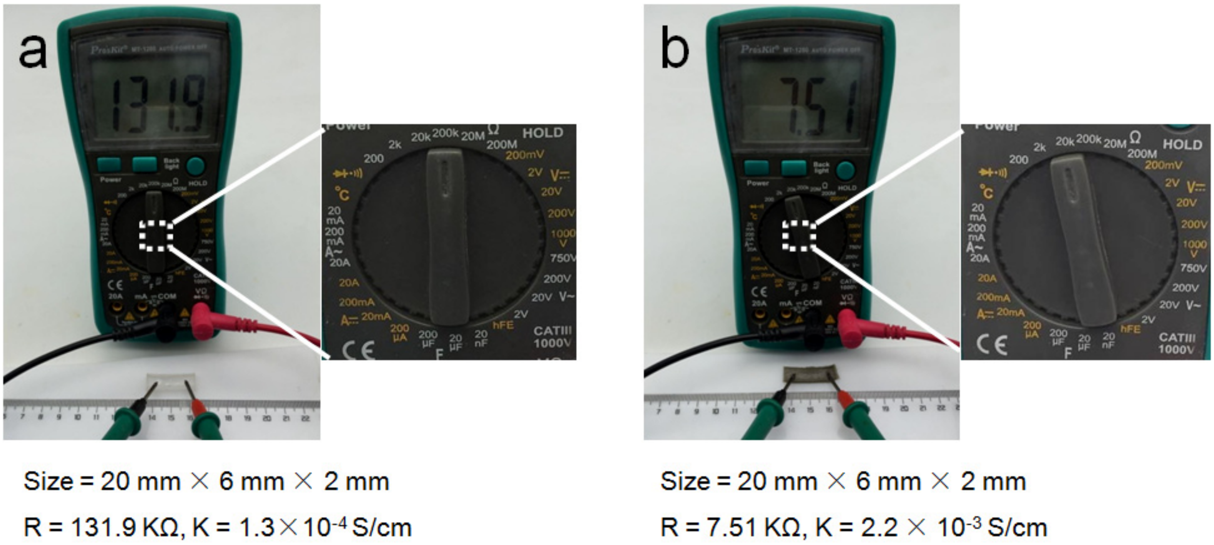

Supplementary Figure 11 | Electrical conductivity of the control hydrogels. Electrical conductivity of **a,** PNIPAM hydrogel and **b,** AgNW/PNIPAM hydrogel. The size of the tested hydrogel was 20 mm x 6 mm x 2 mm.

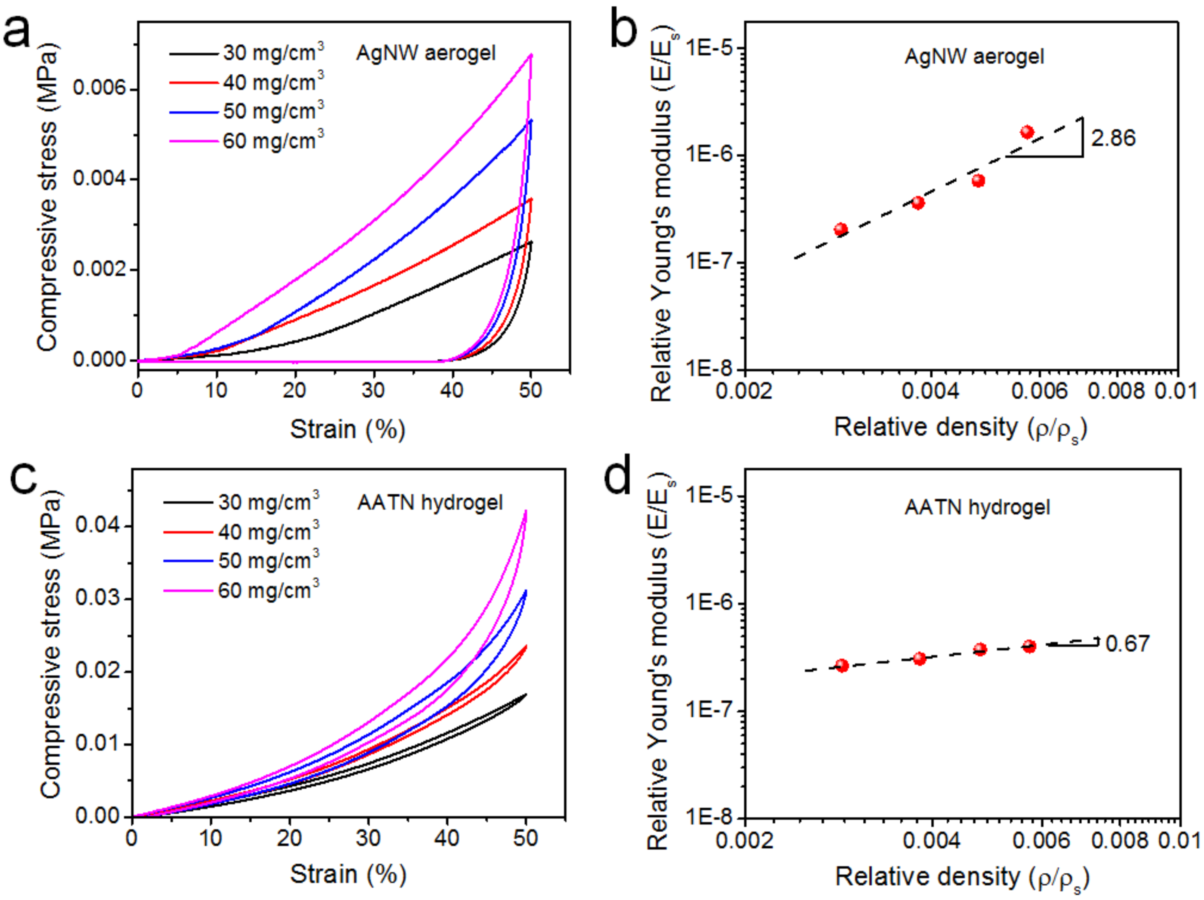

Supplementary Figure 12 | Mechanical properties of AgNW aerogels and AATN hydrogels. Compressive stress-strain curves of **a,** AgNW aerogels and **c,** AATN hydrogels with different contents of AgNWs. The plot of relative Young's modulus (E/E_s) as a function of relative density (ρ/ρ_s) of **b,** AgNW aerogels and **d,** AATN hydrogels.

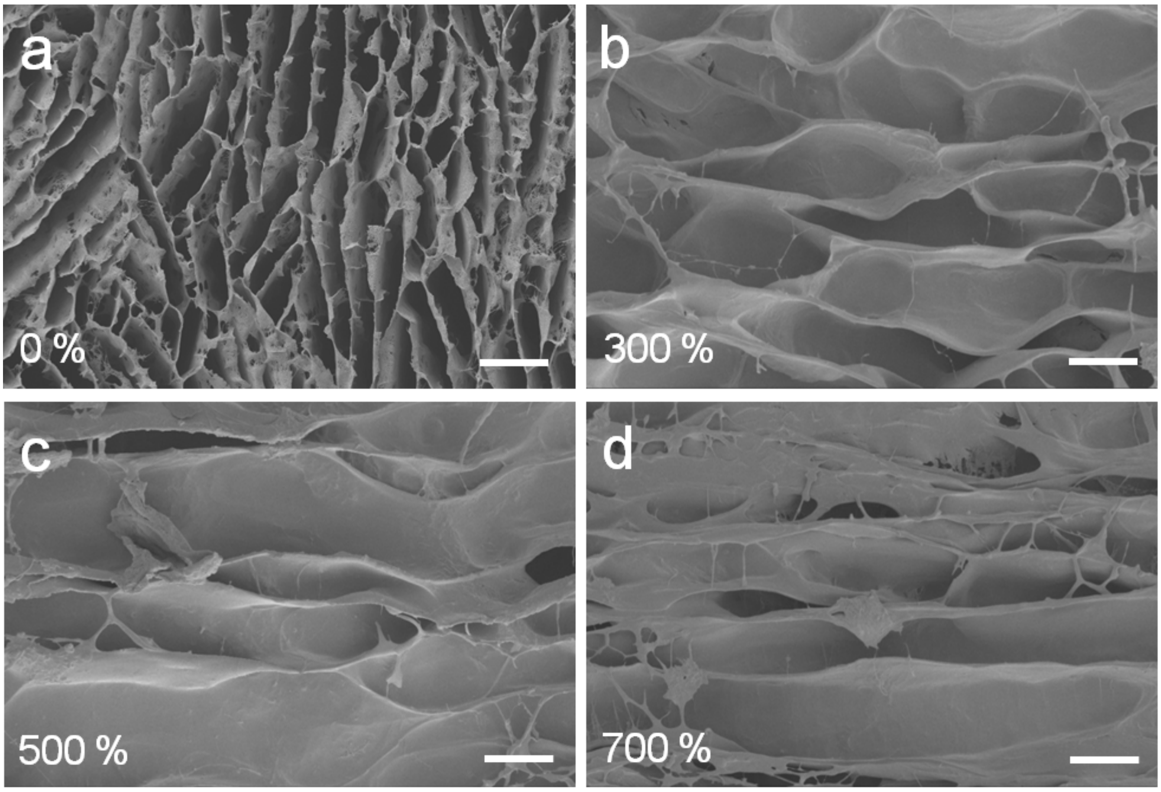

Supplementary Figure 13 | Microstructural evolution of the stretched AATN hydrogel. a-d, Cross-sectional SEM images of AATN hydrogel when stretched with the strain of 0, 300 %, 500 % and 700 %, respectively. *Scale bars* in (**a**) 100 μm and in (**b-d**) 10 μm.

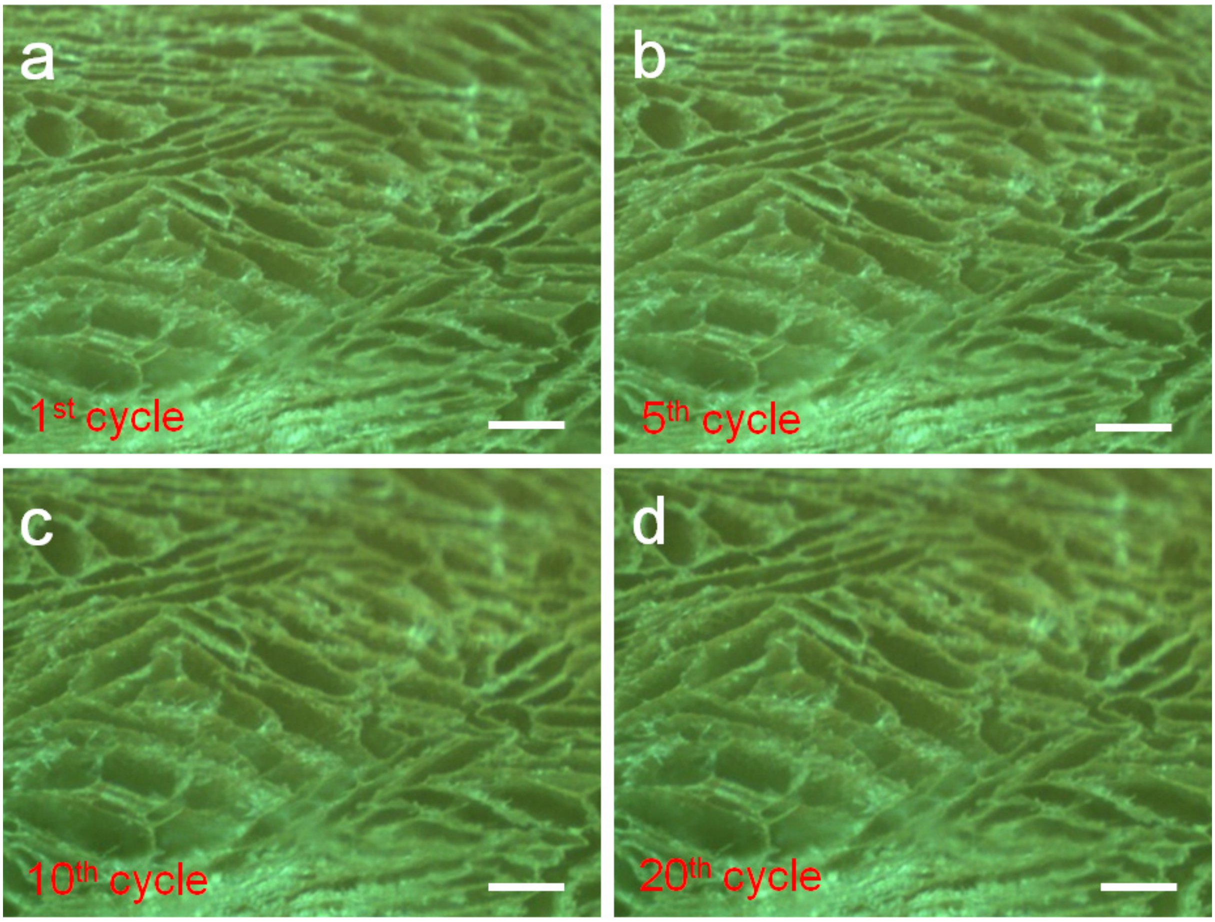

Supplementary Figure 14 | Microstructural evolution of AATN hydrogel during stretching cycles. Optical images of the top-view of AATN hydrogel under a strain of 500% after **a,** 1, **b,** 5, **c,** 10 and **d,** 20 stretching-releasing cycles. *Scale bars* in (**a-d**) 100 µm.

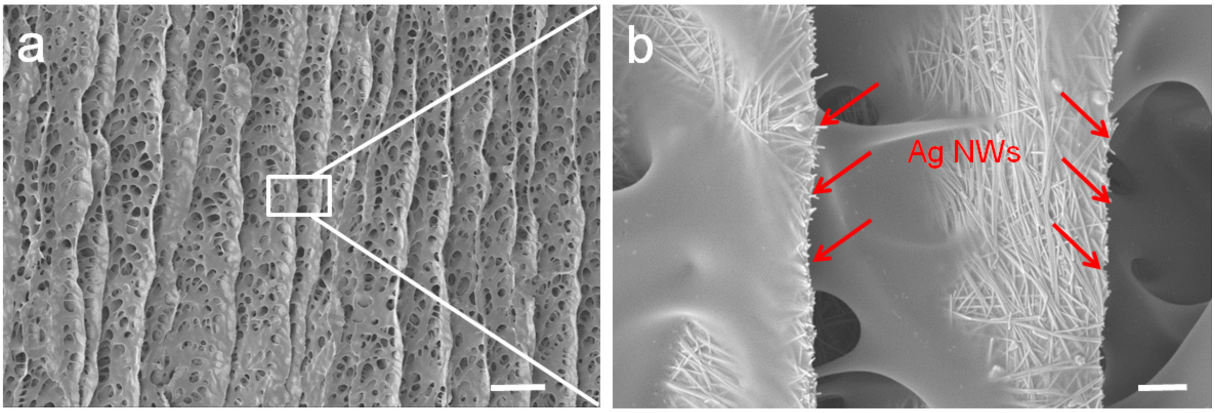

Supplementary Figure 15 | Microstructure of the fractured AATN hydrogel. a, Side-view SEM image of the fractured AATN hydrogel. *Scale bar*, 30 μm. **b,** Magnified SEM image in (**a**). *Scale bar*, 2 μm.

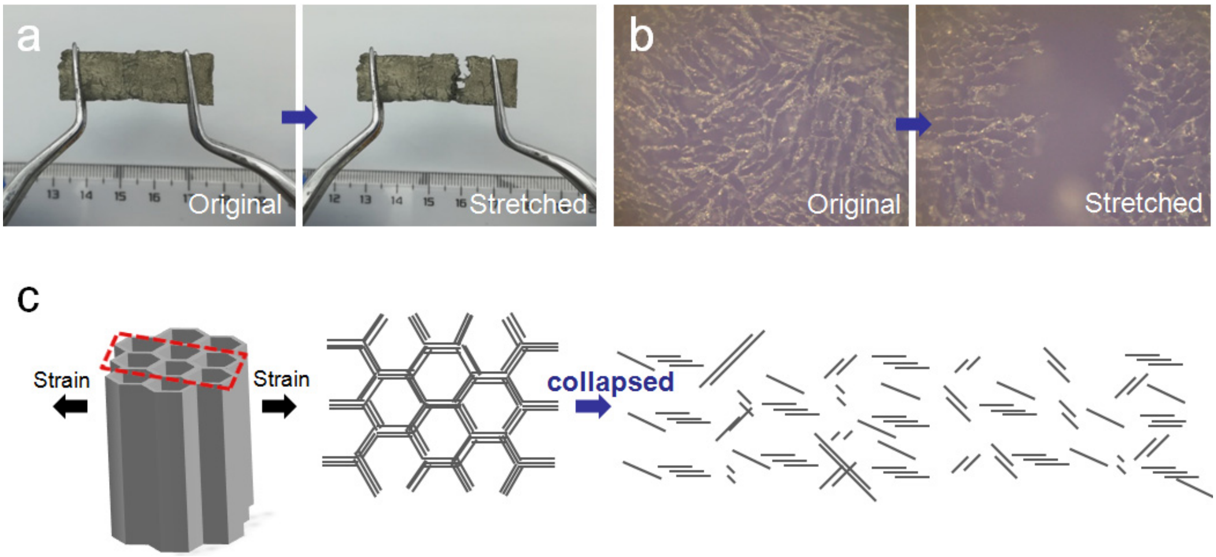

Supplementary Figure 16 | Microstructural analysis of AgNW aerogel under stretching force. a, Photographs of the damaged AgNW aerogel under a small stretching force. **b,** Optical microscopy image of the top view of the damaged AgNW aerogel. **c,** Schematic illustrations of the collapsed network of the AgNW aerogel under a tensile strain.

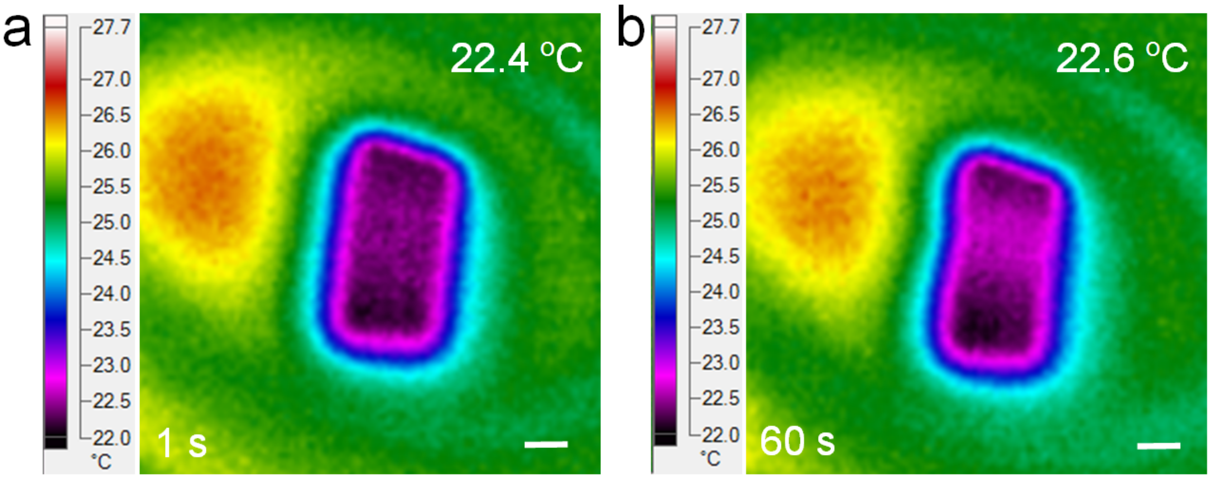

Supplementary Figure 17 | Photothermal property of PNIPAM hydrogel. Temperature changes of PNIPAM hydrogel with the irradiation times of **a,** 0 and **b,** 60 s. *Scale bars* in (**a, b**) 1 cm.

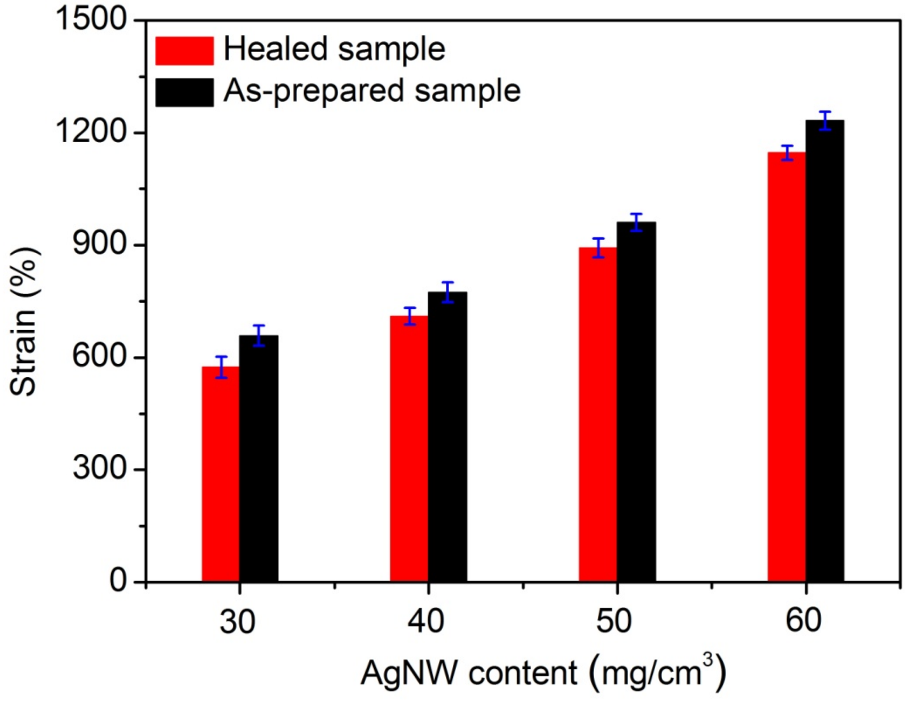

Supplementary Figure 18 | Comparison of strains of a series of the original AATN hydrogels and the healed samples with different AgNW contents.

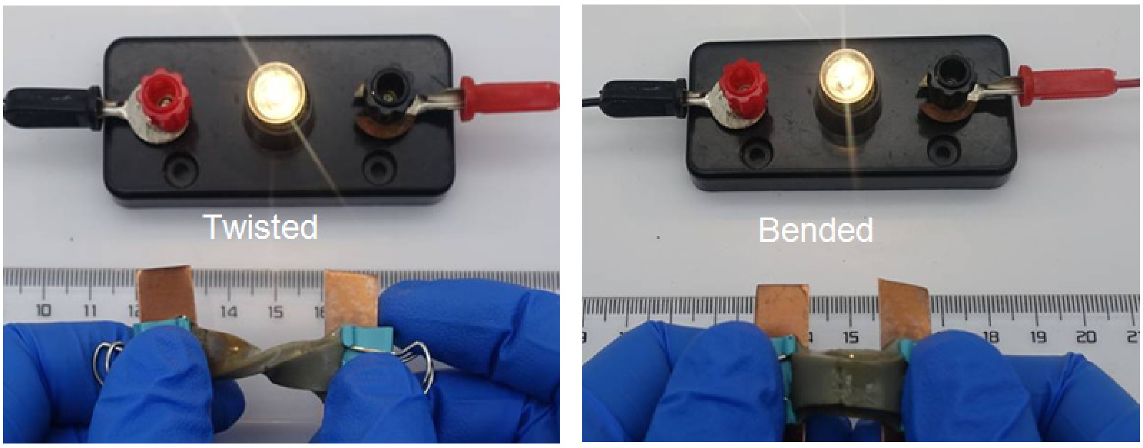

Supplementary Figure 19 | Photographs of the healed AATN hydrogel lightening up the lamp under twisted and bended deformations.

Supplementary Table 1 | Comparison of the electrical conductivity of AATN hydrogel with the reported conductors.

Materials		Conductive constituent content	Electrical conductivity (S/cm)	Reference
CNT-based Conductor	SWCNT aerogel/PDMS films	8-10 mg cm^{-3}	0.7-1.08	1
	SWCNT film/ Dimethylsiloxane-based rubber	20 wt%	57	2
	CNT/PNIPAM	25 wt%	--	3
	MWCNT/PU	5 wt%	0.5-1	4
Graphene-based Conductor	Graphene/PS	0.5 vol%	1×10^{-4}	5
	Graphene hydrogel	2 mg cm^{-3}	5×10^{-3}	6
	Cork-like graphene	5.1 mg cm^{-3}	0.12	7
	rGO/PVA	44 wt%	0.17	8
	Graphene foam/PDMS	5 mg cm^{-3}	10	9
	GrNi/PE	40 vol%	50	10
Metal NW-based Conductor	CuNW aerogel/PVA	10 mg cm^{-3}	0.83	11
	PUS-AgNW-PDMS	20 mg cm^{-3}	10.25	12
	AgNW/PDMS	60 mg cm^{-3}	49.1	13
Others	p-BC/PDMS	< 0.3 vol%	0.2-0.41	14
	PPy/PNIPAM	5 wt%	0.8	15
	SiO_2/Alginate hydrogel	1 wt%	1.85	16
	AATN hydrogel	60 mg cm^{-3}	58	This work

PDMS: Polydimethylsiloxane, PNIPAM: Poly(N-isopropylacrylic amide), PU: Polyurethane, PS: Polystyrene, PVA: Poly(vinyl alcohol), PE: Polyethylene, PUS: Polyurethane sponge, p-BC: Pyrolyzed bacterial cellulose, PPy: Polypyrrole.

Supplementary Table 2 | Comparison of the normalized resistance change ($\Delta R/R_0$) of AATN hydrogel at maximum strain with the reported strechable conductors.

**Materials**		**($\Delta R/R_0$)/ (Maximum strain)**	**Reference**
CNT-based Conductor	SWCNT aerogel/PDMS films	10% / (100%)	1
	SWCNT film/ Dimethylsiloxane-based rubber	-- / (134%)	2
	MWCNT/PU	310% / (300%)	4
	SWCNT/PDMS film	15% / (30%)	17
	SWCNT/PtBA	50% / (50%)	18
	SWCNT/PU-PEDOT:PSS	10^4% / (100%)	19
	SWCNT film/PDMS	-- / (100%)	20
	SWCNT/PDMS	40% / (160%)	21
	CNT/graphene film	-- / (10%)	22
	Wavy CNT ribbon/PDMS	4.1% / (100%)	23
	CNT/PDMS	160% / (100%)	24
	CNT ribbons/PDMS	151% / (220%)	25
	MWCNT/3D-C	900% / (300%)	26
	CNT film/VHB 4905	10^2-10^4% / (100%)<br>10^4-10^5% / (300%)<br>10^5-10^6% / (500%)<br>10^6-10^7% / (700%)	27
Graphene-based Conductor	Graphene/PET	-- / (6%)	28
	Graphene film/PDMS	-- / (30%)	29
	GnPs film/PDMS	250% / (12%)	30
	Graphene-AgNW/Polyimide	20% / (100%)	31
	rGO/elastic tape film	10% / (150%)	32
Metal NW-based	CuNW-PVA-PDMS	19.5% / (60%)	11
	AgNWs/PUS-AgNW-PDMS	160 % / (100%)	12

Conductor	AgNW/PDMS	67 % / (100%)	13
	AgNW film/PDMS	360 % / (85%)	33
	AgNW-AgNP/SBS	-- / (100%)	34
	Zigzag mesh AgNW/PDMS	500% / (125%)	35
	AgNW/PU	400% / (133%)	36
	Ag-MWCNT/1-butyl-4-methylpyridinium tetrafluoroborate	-- / (140%)	37
	AgNW film/Ecoflex	500 % / (460%)	38
	DCY-AgNW/PDMS	-- / (500%)	39
	CuNW/PE	50% / (100%)	40
Others	pBC-PDMS	15% / (80%)	14
	PUF/PEDOT/PDMS	20% / (100%)	41
	AATN hydrogel	20% / (100%)<br>40% / (200%)<br>67% / (300%)<br>97% / (400%)<br>130% / (500%)<br>163% / (600%)<br>210 % / (700%)	This work

PtBA: Poly(tert-butylacrylate), PEDOT: Poly(3,4-ethylenedioxythiophene), PSS: Poly(styrenesulfonate), 3D-C: 3D stacked carbon, PET: Polyethylene terephthalate, SBS: Styrene-butadiene-styrene, GnPs: Graphene platelets, PUF: Polyurethane fiber, DCY: Double-covered yarn.

Supplementary Table 3 | Comparison of the normalized resistance change (ΔR/R_0) of AATN hydrogel at certain stretching strain during stretching cycles with the reported strechable conductors.

Materials		Stretching strain	ΔR/R_0 / *n* cycles	Reference
CNT-based Conductor	MWNT/PU	80%	130% / 100	4
	SWCNT/PDMS film	15%	-- / 100<br>-- / 200	17
	SWCNT/PDMS	50%	～12.5% / 100<br>～12.5% / 500<br>～12.5% / 1000	21
	MWCNT/3D-C	50%	～50% / 100<br>～50% / 500<br>～50% / 1000	26
Metal NW-based Conductor	CuNW-PVA-PDMS	60%	～20% / 100<br>～21% / 1000	11
	PUS-AgNW-PDMS	50%	-- / 100	12
	AgNW/PDMS	50%	～17% / 100<br>～33% / 1000	13
	Zigzag mesh AgNW/PDMS	100%	～60% / 100<br>～60% / 500<br>～60% / 1000	35
	AgNW/PU	50%	< 50% / 100<br>< 100% / 500	36
	DCY-AgNW/PDMS	300%	--/ 100	39
	CuNW/PE	50%	5% / 100<br>5% / 500<br>5% / 1000	40
	AgNW/PU	30%	～300% / 100	42
	AgNW/PUA	30%	100 - 390% / 100<br>150 - 410% / 500<br>160 - 420% / 1000	43
	AgNP/PU	50%	～900% / 100	44
Others	pBC-PDMS	80%	～10% / 100<br>～10% / 500<br>～10% / 1000	14
	PUF/PEDOT/PDMS	50%	10% / 100	41

	AATN hydrogel	100%<br><br>300%<br><br>500%<br><br>700%	57% / 100<br>132% / 500<br>114% / 100<br>248% / 500<br>191% / 100<br>364% / 500<br>304% / 100<br>520% / 500	This Work

PUA: Polyurethane acrylate.

Supplementary Table 4 | Healing efficiency of AATN hydrogels with different contents of AgNWs based on tensile strains before and after healing process.

AgNW content (mg cm^{-3})	Original strain (%)	Healed strain (%)	Healing efficiency (%)
30	658 ± 27	574 ± 28	87
40	779 ± 26	710 ± 22	90
50	970 ± 23	893 ± 25	92
60	1226 ± 24	1146 ± 19	93

Supplementary References

1. Kim, K. H., Vural, M. & Islam, M. F. Single-walled carbon nanotube aerogel-based elastic conductors. *Adv.Mater.* **23**, 2865-2869 (2011).
2. Sekitani, T. *et al.* A rubberlike stretchable active matrix using elastic conductors. *Science* **321**, 1468-1472 (2008).
3. Yang, Z., Cao, Z., Sun, H. & Li, Y. Composite films based on aligned carbon nanotube arrays and a poly(N-isopropyl acrylamide) hydrogel. *Adv.Mater.* **20**, 2201-2205 (2008).
4. Shin, M. K. *et al.* Elastomeric conductive composites based on carbon nanotube forests. *Adv.Mater.* **22**, 2663-2667 (2010).
5. Stankovich, S. *et al.* Graphene-based composite materials. *Nature* **442**, 282-286 (2006).
6. Xu, Y., Sheng, K., Li, C. & Shi, G. Self-assembled graphene hydrogel via a one-step hydrothermal process. *ACS Nano* **4**, 4324-4330 (2010).
7. Qiu, L., Liu, J. Z., Chang, S. L. Y., Wu, Y. & Li, D. Biomimetic superelastic

graphene-based cellular monoliths. *Nat.Commun.* **3**, 1241 (2012).

8. Zhao, N. F. *et al.* Superstretchable nacre-mimetic graphene/poly(vinyl alcohol) composite film based on interfacial architectural engineering. *ACS Nano* **11**, 4777-4784 (2017).
9. Chen, Z. *et al.* Three-dimensional flexible and conductive interconnected graphene networks grown by chemical vapour deposition. *Nat.Mater.* **10**, 424-428 (2011).
10. Chen, Z. *et al.* Fast and reversible thermoresponsive polymer switching materials for safer batteries. *Nat. Energy* **1**, 15009-15016 (2016).
11. Tang, Y., Gong, S., Chen, Y., Yap, L. W. & Cheng, W. Manufacturable conducting rubber ambers and stretchable conductors from copper nanowire aerogel monoliths. *ACS Nano* **8**, 5707-5714 (2014).
12. Ge, J. *et al.* Stretchable conductors based on silver nanowires: Improved performance through a binary network design. *Angew. Chem. Int. Ed.* **52**, 1654-1659 (2013).
13. Gao, H. L. *et al.* Macroscopic free-standing hierarchical 3D architectures assembled from silver nanowires by ice templating. *Angew. Chem. Int. Ed.* **53**, 4561-4566 (2014).
14. Liang, H. W. *et al.* Highly conductive and stretchable conductors fabricated from bacterial cellulose. *NPG Asia Mater.* **4**, e19 (2012).
15. Shi, Y., Ma, C., Peng, L. & Yu, G. Conductive "smart" hybrid hydrogels with PNIPAM and nanostructured conductive polymers. *Adv. Funct. Mater.* **25**, 1219-1225 (2015).
16. Si, Y. *et al.* Ultrahigh-water-content, superelastic, and shape-memory nanofiber-assembled hydrogels exhibiting pressure-responsive conductivity. *Adv. Mater.* **29**, 1700339 (2017).
17. Liu, K. *et al.* Cross-stacked superaligned carbon nanotube films for transparent and stretchable conductors. *Adv. Funct. Mater.* **21**, 2721-2728 (2011).

18. Yu, Z., Niu, X., Liu, Z. & Pei, Q. Intrinsically stretchable polymer light-emitting devices using carbon nanotube-polymer composite electrodes. *Adv. Mater.* **23**, 3989-3994 (2011).

19. Roh, E., Hwang, B. U., Kim, D., Kim, B. Y. & Lee, N. E. Stretchable, transparent, ultra-sensitive and patchable strain sensor for human-machine interfaces comprising a nanohybrid of carbon nanotubes and conductive elastomers. *ACS Nano* **9**, 6252-6261 (2015).

20. Niu, Z. *et al.* Highly stretchable, integrated supercapacitors based on single-walled carbon nanotube films with continuous reticulate architecture. *Adv. Mater.* **25**, 1058-1064 (2013).

21. Cho, D. *et al.* Three-dimensional continuous conductive nanostructure for highly sensitive and stretchable strain sensor. *ACS Appl. Mater. Interfaces* **9**, 17369-17378 (2017).

22. Shi, J. *et al.* Graphene reinforced carbon nanotube networks for wearable strain sensors. *Adv. Funct. Mater.* **26**, 2078-2084 (2016).

23. Xu, F., Wang, X., Zhu, Y. & Zhu, Y. Wavy ribbons of carbon nanotubes for stretchable conductors. *Adv. Funct. Mater.* **22**, 1279-1283 (2012).

24. Zhu, Y. & Xu, F. Buckling of aligned carbon nanotubes as stretchable conductors: A new manufacturing strategy. *Adv. Mater.* **24**, 1073-1077 (2012).

25. Zhang, Y. *et al.* Polymer-embedded carbon nanotube ribbons for stretchable conductors. *Adv. Mater.* **22**, 3027-3031 (2010).

26. Chae, C. *et al.* 3D-stacked carbon composites employing networked electrical intra-pathways for direct-printable, extremely stretchable conductors. *ACS Appl. Mater. Interfaces* **7**, 4109-4117 (2015).

27. Hu, L., Yuan, W., Brochu, P., Gruner, G. & Pei, Q. Highly stretchable, conductive, and transparent nanotube thin films. *Appl. Phys. Lett.* **94**, 161108 (2009).

28. Bae, S. *et al.* Roll-to-roll production of 30-inch graphene films for transparent electrodes. *Nat. Nanotechnol.* **5**, 574-578 (2010).

29. Kim, K. S. *et al.* Large-scale pattern growth of graphene films for stretchable transparent electrodes. *Nature* **457**, 706-710 (2009).

30. Shi, G. *et al.* Highly sensitive, wearable, durable strain sensors and stretchable conductors using graphene/silicon rubber composites. *Adv. Funct. Mater.* **26**, 7614-7625 (2016).

31. Lee, M.-S. *et al.* High-performance, transparent, and stretchable electrodes using graphene-metal nanowire hybrid structures. *Nano Lett.* **13**, 2814-2821 (2013).

32. Xu, J., Chen, J., Zhang, M., Hong, J.-D. & Shi, G. Highly conductive stretchable electrodes prepared by in situ reduction of wavy graphene oxide films coated on elastic tapes. *Adv. Electron. Mater.* **2**, 1600022 (2016).

33. Xu, F. & Zhu, Y. Highly conductive and stretchable silver nanowire conductors. *Adv. Mater.* **24**, 5117-5122 (2012).

34. Lee, S. *et al.* Ag nanowire reinforced highly stretchable conductive fibers for wearable electronics. *Adv. Funct. Mater.* **25**, 3114-3121 (2015).

35. Lee, G. *et al.* Omnidirectionally and highly stretchable conductive electrodes based on noncoplanar zigzag mesh silver nanowire arrays. *Adv. Electron. Mater.* **2**, 1600158 (2016).

36. Kim, A., Ahn, J., Hwang, H., Lee, E. & Moon, J. A pre-strain strategy for developing a highly stretchable and foldable one-dimensional conductive cord based on a Ag

nanowire network. *Nanoscale* **9**, 5773-5778 (2017).

37. Chun, K. Y. *et al.* Highly conductive, printable and stretchable composite films of carbon nanotubes and silver. *Nat. Nanotechnol.* **5**, 853-857 (2010).
38. Lee, P. *et al.* Highly stretchable and highly conductive metal electrode by very long metal nanowire percolation network. *Adv. Mater.* **24**, 3326-3332 (2012).
39. Cheng, Y., Wang, R., Sun, J. & Gao, L. Highly conductive and ultrastretchable electric circuits from covered yarns and silver nanowires. *ACS Nano* **9**, 3887-3895 (2015).
40. Cheng, Y. *et al.* Highly stretchable and conductive copper nanowire based fibers with hierarchical structure for wearable heaters. *ACS Appl. Mater. Interfaces* **8**, 32925-32933 (2016).
41. Duan, S., Wang, Z., Zhang, L., Liu, J. & Li, C. Three-dimensional highly stretchable conductors from elastic fiber mat with conductive polymer coating. *ACS Appl. Mater. Interfaces* **9**, 30772-30778 (2017).
42. Jin, H. *et al.* Enhancing the performance of stretchable conductors for e-textiles by controlled ink permeation. *Adv. Mater.* **29**, 1605848 (2017).
43. Liang, J., Tong, K. & Pei, Q. A water-based silver-nanowire screen-print ink for the fabrication of stretchable conductors and wearable thin-film transistors. *Adv. Mater.* **28**, 5986-5996 (2016).
44. Matsuhisa, N. *et al.* Printable elastic conductors by in situ formation of silver nanoparticles from silver flakes. *Nat. Mater.* **16**, 834-840 (2017).
